# Optimizing spatial specificity and signal quality in fNIRS: an overview of potential challenges and possible options for improving the reliability of real-time applications

**DOI:** 10.3389/fnrgo.2024.1286586

**Published:** 2024-06-05

**Authors:** Franziska Klein

**Affiliations:** ^1^Biomedical Devices and Systems Group, R&D Division Health, OFFIS - Institute for Information Technology, Oldenburg, Germany; ^2^Department of Psychiatry, Psychotherapy and Psychosomatics, Medical School, RWTH Aachen University, Aachen, Germany; ^3^Neurocognition and Functional Neurorehabilitation Group, Department of Psychology, University of Oldenburg, Oldenburg, Germany

**Keywords:** fNIRS, real-time preprocessing, neurofeedback, BCI, noise reduction, extracerebral systemic activity, motion artifacts, spatial specificity

## Abstract

The optical brain imaging method functional near-infrared spectroscopy (fNIRS) is a promising tool for real-time applications such as neurofeedback and brain-computer interfaces. Its combination of spatial specificity and mobility makes it particularly attractive for clinical use, both at the bedside and in patients' homes. Despite these advantages, optimizing fNIRS for real-time use requires careful attention to two key aspects: ensuring good spatial specificity and maintaining high signal quality. While fNIRS detects superficial cortical brain regions, consistently and reliably targeting specific regions of interest can be challenging, particularly in studies that require repeated measurements. Variations in cap placement coupled with limited anatomical information may further reduce this accuracy. Furthermore, it is important to maintain good signal quality in real-time contexts to ensure that they reflect the true underlying brain activity. However, fNIRS signals are susceptible to contamination by cerebral and extracerebral systemic noise as well as motion artifacts. Insufficient real-time preprocessing can therefore cause the system to run on noise instead of brain activity. The aim of this review article is to help advance the progress of fNIRS-based real-time applications. It highlights the potential challenges in improving spatial specificity and signal quality, discusses possible options to overcome these challenges, and addresses further considerations relevant to real-time applications. By addressing these topics, the article aims to help improve the planning and execution of future real-time studies, thereby increasing their reliability and repeatability.

## 1 Introduction

Real-time analyses are crucial for neurofeedback (NFB) and brain-computer interface (BCI) applications because they allow instantaneous interpretation of brain signals (Enriquez-Geppert et al., 2017; Lührs and Goebel, 2017). In contrast to offline analysis, real-time analysis faces the challenge of maintaining consistent and fast calculation times, while ensuring that incoming data are continuously processed within these limited time frames (Lührs and Goebel, 2017). This allows for immediate feedback and control. BCIs and NFB rely on brain imaging data obtained using techniques such as electroencephalography (EEG), functional magnetic resonance imaging (fMRI), or functional near-infrared spectroscopy (fNIRS), and their real-time data processing includes data streaming, (pre-)processing, and feedback generation (Naseer and Hong, 2015; Enriquez-Geppert et al., 2017; Sitaram et al., 2017; Mahrooz et al., 2023). Despite certain methodological similarities, there are different definitions of BCI and NFB depending on the source or expert, particularly regarding their areas of application (Mahrooz et al., 2023). BCIs are often described as systems that enable direct communication between the brain and external devices, allowing individuals to interact with their environment using their own brain signals (Naseer and Hong, 2015; Paulmurugan et al., 2021; Saha et al., 2021; Mahrooz et al., 2023). BCIs are used in different fields such as assistive technology, neuroprosthetics, and entertainment (Millán, 2010; Rupp et al., 2014; Abdulkader et al., 2015). On the other hand, NFB focuses more on self-regulation of brain activity by providing feedback through sensory cues with the overall goal of improving cognitive functions or alleviating symptoms (Marzbani et al., 2016; Sitaram et al., 2017; Kohl et al., 2020; Soekadar et al., 2021). Accordingly, NFB is more commonly used in clinical settings, and research on this topic includes conditions such as ADHD (e.g., Hudak et al., 2018; Rubia et al., 2021), anxiety (e.g., Kimmig et al., 2019; Linhartová et al., 2019; Lipp and Cohen Kadosh, 2020), depression (e.g., Trambaiolli et al., 2021; González Méndez et al., 2022), stroke (e.g., Rieke et al., 2020; Sanders et al., 2022), and Parkinson's disease (e.g., Subramanian et al., 2011, 2016; Mehler, 2022). Because of the methodological overlap, BCI and NFB probably cannot be considered as completely independent methods. Since both applications involve some kind of feedback mechanism, NFB could be viewed as a specific form of BCI method that focuses on (therapeutic) training and self-regulation of brain activity (Mahrooz et al., 2023).

Real-time hemodynamic-based BCIs and NFB applications have been shown to complement traditional electrophysiological-based methods such as EEG. Reflecting the changes in blood flow and oxygenation of the brain, hemodynamic signals are captured using methods such as fMRI and fNIRS and provide valuable insights into brain activity (Sitaram et al., 2009; Naseer and Hong, 2015; Mihara and Miyai, 2016; Wang et al., 2018; Kohl et al., 2020; Paulmurugan et al., 2021; Pindi et al., 2022). For instance, fNIRS captures hemodynamic brain signals using optodes (i.e., light sources and detectors) placed on the head surface. It measures changes in the absorption of near-infrared light and reflects changes in concentration in oxygenated (Δ[*HbO*]) and deoxygenated (Δ[*HbR*]) hemoglobin as the light travels from the source to the detector (Scholkmann et al., 2014; Pinti et al., 2020). While fMRI, considered the gold standard in hemodynamic brain imaging, provides whole-brain measurements, fNIRS has limitations in terms of spatial resolution as it can only access the superficial cortex (Scarapicchia et al., 2017; Klein et al., 2022a). However, fNIRS offers several advantages compared to fMRI. It is more cost-effective and it allows the inclusion of different populations without specific method-based exclusion criteria. Furthermore, because fNIRS measures both Δ[*HbR*] and Δ[*HbO*], it offers insights into hemodynamics and tissue oxygenation and therefore provides more information than the fMRI blood-oxygen-level-dependent (BOLD) signal, which reflects the Δ[*HbR*] only. In addition, fNIRS, with its superior temporal resolution (typically ~10 Hz compared to ~1 Hz for fMRI), better distinguishes between higher frequency activities such as cardiac (~1 Hz) and respiratory activities (~0.3 Hz) and the lower frequency signals of interest. Finally, fNIRS has lower sensitivity to motion-related artifacts and offers portable and mobile measurements, enabling measurements in real-world environments, from bedside applications in the hospital to the home or virtually any other location (Cui et al., 2011; Scarapicchia et al., 2017; Machado et al., 2018; Quaresima and Ferrari, 2019; Pinti et al., 2020; Von Lühmann et al., 2021; Klein et al., 2022a,b; Scholkmann et al., 2022). These advantages make fNIRS particularly interesting for real-time applications (Lu et al., 2010; Kohl et al., 2020; Soekadar et al., 2021).

fNIRS is a rapidly evolving technique with many possibilities for offline and real-time applications. Methodologically, the field of fNIRS-based real-time applications lags behind that of offline applications, which could be due, among other things, to a lack of comprehensive recommendations and strict validation processes. This deficiency can complicate the interpretation, comparison, and replication of fNIRS studies, a problem already identified in the fNIRS community. For instance, the lack of standardized analysis pipelines is a major problem which can be further complicated by inadequate reporting practices, thereby significantly reducing the impact, replicability, and reproducibility of published results (Kohl et al., 2020; Yücel et al., 2021; Kelsey et al., 2023; Schroeder et al., 2023). The importance of strict adherence to methodological standards and research practices is underscored by a recent incident of scientific misconduct in which an fNIRS-based BCI was tested for communication with patients with degenerative diseases such as amyotrophic lateral sclerosis (Chaudhary et al., 2017, 2019; Spüler, 2019). An investigation of the published study revealed several issues, including selective omission of data without transparent criteria, lack of disclosure of data and analysis scripts, discrepancies between reported and available data, and potential data bias due to incorrect data analysis. These results led to recommendations for the study's withdrawal and highlighted the urgent need for transparency and careful methodology in research in real-time analysis and beyond.^1^

Two challenges in particular should be highlighted in the context of fNIRS-based real-time applications: improving spatial specificity and signal quality. Due to limited anatomical information and typically low head coverage, it is challenging to achieve precise and consistent spatial targeting of relevant regions of interest (ROIs). However, in applications such as NFB, repeatability of optode placement is critical for reliably training specific ROIs across multiple sessions (Benitez-Andonegui et al., 2021; Klein et al., 2022a). Similarly, ensuring sufficient signal quality is important. Insufficient real-time preprocessing in fNIRS can lead to a system that operates on noise rather than brain activity (Klein et al., 2022b), which might not only reduce the effectiveness of the application itself, but also could undermine the user's trust in the system. Since real-time processing, unlike offline analysis, does not allow corrections after data acquisition, the use of effective and robust real-time preprocessing techniques is even more important to extract meaningful signals and improve the accuracy of measurements to ensure reliability in real-time applications.

To address this gap, the aim of this review is to identify potential challenges in fNIRS applications regarding spatial specificity and signal quality and discuss these for their potential application in real-time scenarios. Since systematic methodological validations and reviews for real-time contexts are still very limited, this work discusses offline methods that are considered potentially suitable for real-time applications based on the authors' individual knowledge and experiences. Please note, however, that the selection of methods is not exhaustive and may not cover all possible options. For this purpose, this review is divided into two main sections: *Improvement of spatial specificity* (see Section 2) and *Improvement of signal quality* (see Section 3). Each section explores various subtopics, outlines potential challenges, suggests possible options for improvement strategies, and explores further considerations for their applicability in the context of fNIRS-based real-time applications. Please note that the figures in the manuscript showing data comparing different signal processing methods are for illustrative purposes only and come from individual, partly unpublished data sets and do not reflect group analyses. They are intended solely as a visual aid and not for in-depth analytical interpretation. Additionally, it is important to note that these figures represent offline preprocessed data, as there is currently a lack of freely available tools for real-time processing. Finally, it is important to note that the option boxes in each of the *Possible Options* sections are intended to highlight and complement the main text. Looking at these boxes in isolation could disrupt the logical reading flow.

## 2 Improving spatial specificity

**Figure 1:**
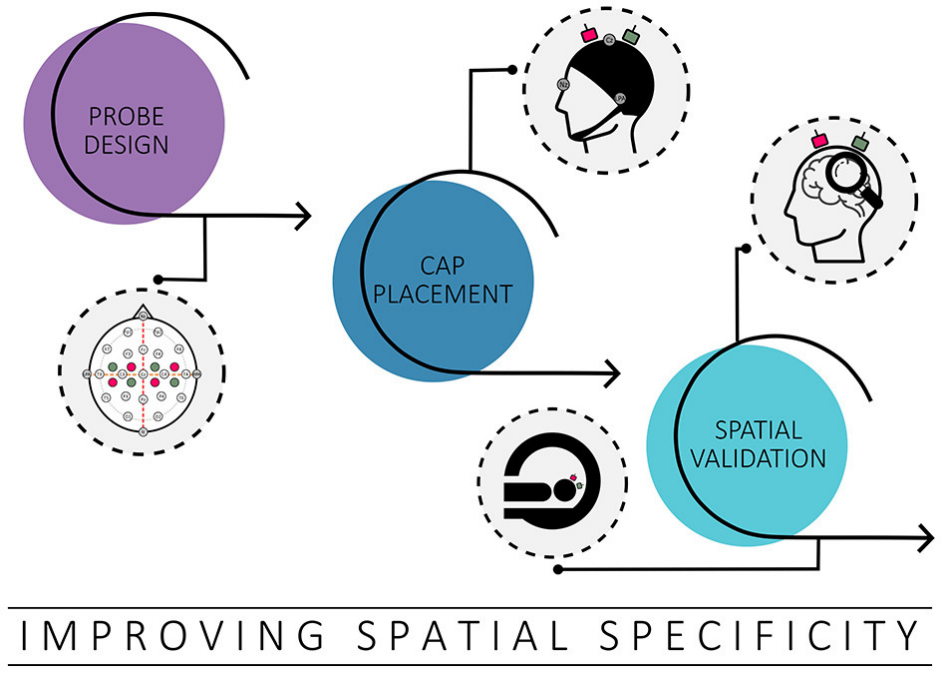
Overview of strategies covered to improve spatial specificity. Possible options discussed in this context include probe design, cap placement and spatial validation.

fNIRS offers good spatial specificity for superficial cortical brain regions (Scarapicchia et al., 2017; Pinti et al., 2020). However, achieving accurate ROI coverage can be challenging, especially with a limited number of optodes and a lack of individual anatomical information. The complexity is further compounded by the large functional and structural variability between individual brains (Uylings et al., 2005; Van Horn et al., 2008; Duffau, 2017). This issue of inter-individual variability highlights the need for improved and potentially more individualized approaches in fNIRS experiments to increase spatial specificity as well as measurement accuracy. Initial steps toward improvement include effective probe design, accurate cap placement, and validating the ability of fNIRS to target specific ROIs, for instance, by combining fNIRS data with higher-resolution brain imaging techniques such as fMRI (cf. Figure 1) (Toronov et al., 2001; Strangman et al., 2002; Cui et al., 2011; Noah et al., 2015; Abdalmalak et al., 2017; Huppert et al., 2017; Brigadoi et al., 2018; Zimeo Morais et al., 2018; Klein et al., 2022a). Appropriate implementation can improve the reliability of fNIRS measurements, especially in real-time applications where repeated precise targeting is critical to obtain reliable results (Klein et al., 2022a).

### 2.1 Probe design

#### 2.1.1 Potential challenges

Designing an appropriate probe layout is a fundamental step for performing fNIRS experiments. However, designing a probe layout that achieves optimal coverage of the ROI can be challenging, especially with limited available optodes and the lack of individual anatomical information (Brigadoi et al., 2018; Zimeo Morais et al., 2018). In cases where individual anatomical information is not available, researchers often rely on standard templates or probabilistic atlases to guide optode placement (Brigadoi et al., 2018; Zimeo Morais et al., 2018). In addition, factors such as age, head circumference, and anatomical differences between different populations (e.g., due to differences in race/ethnicity) could influence probe design. Therefore, cross-study probe designs, even when targeting the same ROI, may not be directly applicable due to individual variations and may not be appropriate for different populations (Farkas et al., 1992; Bastir et al., 2006; Brigadoi et al., 2018). Accordingly, careful consideration is required to address these challenges.

#### 2.1.2 Possible options

To ensure reproducibility and consistent cap placement, it is recommended to design the probe layout in relation to standardized landmarks such as the international 10–20 or 10–5 EEG reference system (Oostenveld and Praamstra, 2001; Jurcak et al., 2007; Brigadoi et al., 2018; Zimeo Morais et al., 2018). There are several tools available that can use these standardized EEG positions as a basis for designing probe layouts and/or quantify them through so-called photon transport simulations (PTS) (Fang and Boas, 2009; Tran et al., 2020). For instance, PTS can be used to create sensitivity profiles for each channel of a probe design to estimate its sensitivity to a specific ROI (Aasted et al., 2015; Brigadoi et al., 2018; Zimeo Morais et al., 2018; Fu and Richards, 2021). A user-friendly option for designing a probe layout is the MATLAB-based toolbox called fOLD (*fNIRS Optodes' Location Designer*; Zimeo Morais et al., 2018).

**fNIRS Optodes' Location Designer (fOLD)**

fOLD (https://github.com/nirx/fOLD-public; Zimeo Morais et al., 2018) is an easy-to-use tool that helps design probe layouts to target specific ROIs. Based on various brain parcellation atlases, possible optode positions can be determined based on standard EEG positions (Zimeo Morais et al., 2018). Because fOLD is restricted to adult populations, a derivation of fOLD called devfOLD was developed for researchers studying infants (*developmental fNIRS Optodes' Location Designer*; https://github.com/nirx/devfOLD; Fu and Richards, 2021).

While the use of fOLD contributes to standardization and replicability in fNIRS research, it may not account for individual anatomical differences or provide individual information on channel sensitivity (Zimeo Morais et al., 2018). An alternative MATLAB-based toolbox that is particularly useful for evaluating the sensitivity of a probe layout is AtlasViewer (Aasted et al., 2015).

**AtlasViewer**

While AtlasViewer (https://github.com/BUNPC/AtlasViewer) offers general probe design capabilities, it is primarily based on a more subjective point-and-click approach and does not have an objective ROI-based design capability for probe layouts like fOLD (Brigadoi et al., 2018). However, AtlasViewer has an advantage over fOLD/devfOLD as it allows quantification of the sensitivity of a previously designed probe layout. By loading 3D channel locations into the toolbox, sensitivity profiles can be generated based on standard head models or individual anatomy. This enables researchers to evaluate whether the probe layout sufficiently targets the ROI. To perform such validation, additional information on predefined individual anatomical landmarks including nasion (Nz), inion (Iz), left and right preauricular points (LPA and RPA), as well as Cz are required (Aasted et al., 2015).

AtlasViewer is a widely used and flexible tool for evaluating the sensitivity of probe layouts. However, the pure probe design process can become time-consuming due to subjective decision-making (Brigadoi et al., 2018).

Another way to design probe layouts is to use the Array Designer toolbox (Brigadoi et al., 2018).

**Array Designer**

Array Designer (https://github.com/DOT-HUB/ArrayDesigner; Brigadoi et al., 2018) can be used to generate sensitivity profiles for a variety of possible channel locations based on a standard head model or individual anatomy. The user can set parameters such as the number of available optodes and the desired minimum and maximum distance between sources and detectors. The toolbox allows ROIs to be defined by either selecting them from an available atlas, entering MNI coordinates, or manually selecting the ROIs using a point-and-click approach (Brigadoi et al., 2018). Based on the given specifications, Array Designer identifies the optimal solution to cover the ROIs, labels each optode with a 10–5 position (or 10–2.5 position) and provides information on the total sensitivity in millimeters, percentage of ROI coverage as well as the average, minimum and maximum distance between source and detector (Brigadoi et al., 2018).

As a flexible and automated tool, Array Designer offers a way to design reproducible fNIRS probe layouts (Brigadoi et al., 2018). However, a limitation highlighted by the authors is that the underlying optimization algorithm used in Array Designer may not always yield the optimal solution, especially when working with large optode arrays (Brigadoi et al., 2018).

Compared to fOLD, both AtlasViewer and Array Designer offer the ability to integrate individual anatomical information into the probe design process, allowing researchers to improve spatial specificity, potentially helping to improve the precision and reliability of fNIRS measurements. To further increase accuracy, additional individual information could be taken into account (Benitez-Andonegui et al., 2021).

**Adding More Individual Information**

More specifically, the precision and accuracy could be further improved by incorporating additional individual information such as functional and vascular data (Benitez-Andonegui et al., 2021). By including such data, probe design could be optimized to account for between-subject variability, resulting in more precise and robust measurements in fNIRS studies. For instance, functional information could explain differences in activation patterns across individuals. In addition, the properties of individual vascular information could be highly relevant for assessing light sensitivity due to their strong scattering and absorption properties, which makes the individual analysis even more complex (Benitez-Andonegui et al., 2021).

#### 2.1.3 Further considerations

A study by Benitez-Andonegui et al. (2021) compared different probe design approaches to find out which approach provides the most added value in BCI experiments. They compared a literature-based design with fOLD, the use of functional and anatomical information from an independent data set, the use of individual anatomical and functional information, and the use of individual anatomical, functional and vascular information. The study found that probe designs based on MRI information outperformed designs generated by fOLD. However, given the trade-offs between time, cost, and improvement, the authors suggested that general anatomical information is sufficient and that anatomy does not necessarily have to come from the individual in order to achieve better results (Benitez-Andonegui et al., 2021). The authors recommended using individual functional and anatomical data whenever possible when designing optode layouts. In cases where only anatomical data is accessible, probabilistic functional maps could be a promising and cost-effective replacement (Benitez-Andonegui et al., 2021).

If no individual anatomical information is available, tools like fOLD and Array Designer appear to be a promising option as they can facilitate the creation of reproducible probe layouts. Using Array Designer or AtlasViewer, layouts can be adapted to individual anatomies or general head models and evaluated using PTS (Aasted et al., 2015; Brigadoi et al., 2018; Zimeo Morais et al., 2018). Regardless of the specific software used for probe design, accurate reporting of design details in research publications is critical to a study's reproducibility. Comprehensive documentation should include information on the number and placement of optodes, the targeted ROI, any relevant anatomical considerations or landmarks, and the validation methods used (Yücel et al., 2021). Transparent reporting plays an important role in improving reproducibility by enabling accurate replication of experimental conditions.

### 2.2 Cap placement

#### 2.2.1 Potential challenges

In addition to the probe design, the exact placement of the cap is another important prerequisite for carrying out fNIRS experiments, as it contributes significantly to the precision and reproducibility of the measurements (Novi et al., 2020a). Novi et al. (2020a) emphasized the importance of precise cap placement and stressed that while the reproducibility of fNIRS analysis is generally satisfactory at the group level, it is observed less frequently at the individual level. This result is particularly relevant for real-time applications, as these typically require repeated measurements at the individual subject level.

#### 2.2.2 Possible options

A commonly used approach to probe placement comes from the EEG field and involves standardized positioning of the cap based on established systems such as the international 10–20, 10–10 or 10–5 EEG system (Klem et al., 1999; Oostenveld and Praamstra, 2001).

**Standardized 10–20 Cap Placement**

**Figure 2:**
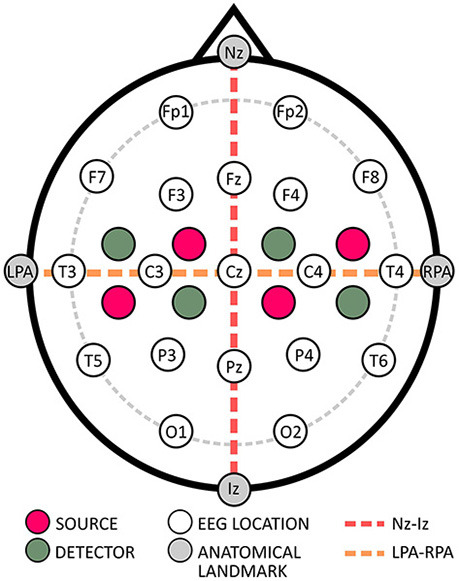
Exemplary probe placement relative to the 10–20 EEG system.

An important reference point for the placement of caps within the standardized EEG systems is Cz, whose exact placement occurs at the vertex, which lies where the Nz to Iz and LPA to RPA lines intersect (cf. Figure 2). Typically, this intersection point is at 50% of the length of the respective lines (Klem et al., 1999; Oostenveld and Praamstra, 2001). To ensure the cap is properly placed, it is recommended to inspect the cap from the front and back to verify symmetrical alignment both horizontally and vertically, effectively controlling any shifts (Klem et al., 1999; Oostenveld and Praamstra, 2001).

The advantage of this standardized approach is that consistent and repeatable cap placement could be improved by reducing different cap placement strategies, which can lead to increased variability both within and between subjects, could be reduced. However, the standardized approach could also be difficult to follow, for instance, when individuals do not have a tactile Iz landmark. Moreover, the standard approach does not account for inter-individual anatomical differences, potentially leading to sub-optimal placements. To address these issues, the real-time neuronavigator approach has been presented as a promising alternative (Novi et al., 2020a; Wu et al., 2021).

**Neuronavigator Approach**

The neuronavigator approach is based on the use of neuronavigation software used in transcranial magnetic stimulation to control coil placement. This approach requires an individual anatomical image and a digitizing system to guide and ensure correct placement of the cap (https://www.dca.fee.unicamp.br/projects/mtk/rubianesD/downloads.html; Novi et al., 2020a; Wu et al., 2021). By registering each 3D optode position on the participant's head and aligning it with the anatomical image, a real-time 3D view is created, allowing for individual adjustments to cap placement (Novi et al., 2020a; Wu et al., 2021).

Overall, the neuronavigator approach has the potential to improve anatomically accurate cap placement (Novi et al., 2020a; Benitez-Andonegui et al., 2021; Wu et al., 2021). However, this approach appears to be based exclusively on purely anatomical MRI data and does not offer the possibility of selecting brain regions from a brain atlas, for example. Therefore, additional individual functional information is required to effectively guide cap placement. Moreover, this method requires additional hardware, which increases the complexity and overall cost of the process.

#### 2.2.3 Further considerations

Using the neuronavigator approach, it was shown that the within-subject reproducibility of fNIRS measurements can be increased (Novi et al., 2020a). Thus, when factors such as cost and time are not a constraint, the neuronavigator approach can be a valuable tool for improved cap placement in real-time experiments. However, when individual anatomical data is unavailable or difficult to obtain, the standard approach to cap placement could be considered. Adherence to standardized approaches is critical to ensure reproducibility of fNIRS measurements within subjects and across different studies. Compliance with such standards can help promote consistency, comparability and transparency. Moreover, in order to be able to reproduce the corresponding experimental setup exactly, it is also important to report the exact procedure for cap placement (Yücel et al., 2021).

### 2.3 Spatial validation

#### 2.3.1 Potential challenges

Since the spatial resolution of fNIRS is ~2–3 cm and the depth resolution is ~1.5–2 cm, it mainly detects activity from the superficial cortical layers of the brain, thus showing good spatial specificity to the activity from the regions directly beneath the optodes (Pinti et al., 2020). However, due to the different layer thicknesses between the scalp and different brain regions, challenges may arise that potentially affect the accuracy of fNIRS measurements in certain ROIs (Cui et al., 2011). Therefore, if the goal is to target a ROI that may be difficult to reach, it might be beneficial to first validate whether fNIRS can reliably capture activity from that specific ROI (Klein et al., 2022a). Such validation processes could help ensure the suitability of fNIRS for achieving intended ROIs, thereby providing greater confidence in the reliability and interpretability of the results.

#### 2.3.2 Possible options

A possible option to ensure reliable fNIRS measurements is to conduct a validation study using fMRI, as this method is considered the gold standard for spatially specific brain imaging (Cui et al., 2011; Klein et al., 2022a). In such a study, coregistration analysis incorporating individual anatomical information and registered 3D optode positions can be performed to assess the spatial specificity of the fNIRS measurements (Aasted et al., 2015; Klein et al., 2022a).

**Coregistration**

The general goal of a coregistration is to align anatomical images with 3D optode positions using common anatomical landmarks such as Nz, LPA and RPA (Cui et al., 2011; Klein et al., 2022a). This alignment can be achieved, for instance, by using the transformation equation (Arun et al., 1987)

$$\begin{array}{l} B=RA+t \end{array}$$

Here, *B* denotes the data that has undergone a transformation to the target space (e.g., head space) while *R* represents the rotation matrix, *A* corresponds to the original data in source space (e.g., optode positions) and *t* denotes the translation vector. This transformation enables the calculation of channel positions on the head surface, which can be used for various purposes, such as creating sensitivity maps using techniques such as PTS (e.g., Abdalmalak et al., 2017; Huppert et al., 2017), or projecting the positions onto the cortex for extracting channel-specific spatial voxel-based information (Cui et al., 2011; Klein et al., 2022a).

By incorporating coregistration processes, such as those offered by tools like AtlasViewer and Nirstorm, (https://github.com/Nirstorm/nirstorm), a plugin for the MATLAB-based software Brainstorm (Tadel et al., 2011), into the planning and validation of real-time interventions, the accuracy of fNIRS measurements could potentially be improved. This is particularly true for applications where it is crucial to understand the brain region's responses to specific tasks (Klein et al., 2022a). These tools facilitate fMRI-fNIRS coregistration by segmenting anatomical images into different layers and converting them into mesh representations (Tran et al., 2020), which are then used to extract coordinates for functional MRI data. This approach can help validate fNIRS measurements for task sensitivity and investigate the relationship between fNIRS signals (e.g., Δ[*HbO*] and Δ[*HbR*]) and BOLD signal for specific tasks and ROIs (Cui et al., 2011; Klein et al., 2022a).

#### 2.3.3 Further considerations

In the earlier days of fNIRS research, coregistration studies were common to validate the reliability of fNIRS measurements across different tasks and brain regions (Toronov et al., 2001, 2003; Mehagnoul-Schipper et al., 2002; Strangman et al., 2002; Cui et al., 2011). Although it appears that their frequency has decreased over time, significant advances in hardware-based correction methods in recent years, such as short-distance channel correction (see Section 3), validation studies should continue to be carried out to extend previous results. Interestingly, there has recently been renewed interest in fMRI-fNIRS validation studies (Abdalmalak et al., 2017; Wagner et al., 2021; Klein et al., 2022a; Novi Junior et al., 2023; Pereira et al., 2023). Although the simultaneous acquisition of (f)MRI and fNIRS data offers optimal conditions (Toronov et al., 2001, 2003; Mehagnoul-Schipper et al., 2002; Strangman et al., 2002; Cui et al., 2011; Anwar et al., 2016; Huppert et al., 2017), it presents technical challenges due to additional hardware requirements. However, as commercially available hardware enabling simultaneous fMRI and fNIRS measurements becomes more accessible (e.g., NIRx Borealis https://nirx.net/borealis; NIRx Medizintechnik, Berlin, Germany), it is possible that this trend will increase again. Alternatively, sequential designs are also used in which fMRI and fNIRS data are acquired separately (Noah et al., 2015; Abdalmalak et al., 2017; Klein et al., 2022a; Pereira et al., 2023).

Conducting validation studies before developing fNIRS-based real-time applications is helpful to verify the accuracy of fNIRS in measuring target ROIs, identify effective tasks for activating these ROIs, and determine the most sensitive signal type for each task-ROI combination (Klein et al., 2022a). Despite the additional time and cost, these validation studies represent a promising opportunity to improve task sensitivity and spatial specificity in real-time applications. The findings from these studies are applicable not only to the specific application being validated, but also to future use cases with similar task-ROI combinations (Klein et al., 2022a).

## 3 Improving signal quality

**Figure 3:**
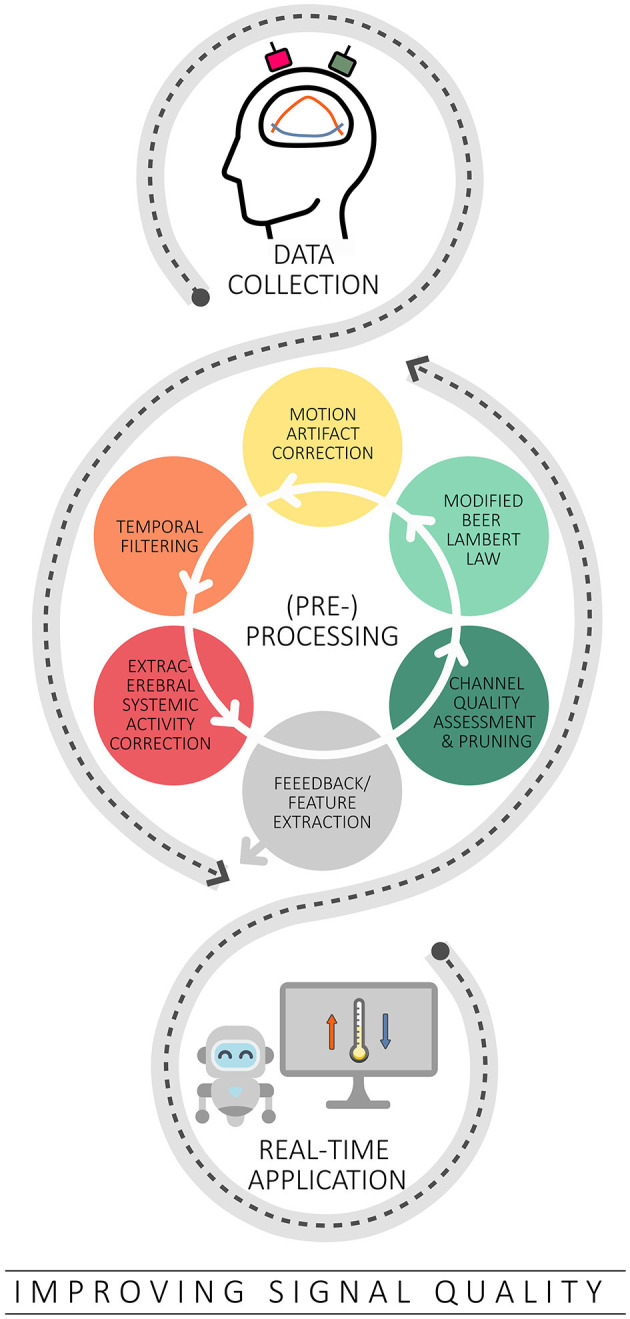
Overview of the strategies covered to improve signal quality in the context of real-time applications. Based on typical offline methods, channel quality assessment and correction, the modified Beer Lambert law, correction of motion artifacts, temporal filtering and correction of extracerebral systemic artifacts are discussed in this context.

In addition to efforts to improve the spatial specificity of fNIRS, another important area of improvement is the quality of the fNIRS signal, as it can be affected by a variety of noise sources. One source of noise is motion artifacts (MAs), which are mainly caused by body and head movements and can lead to distortions in the acquired fNIRS signal (Pollonini et al., 2016). At the same time, systemic activity noise—including task-evoked and non-evoked activities in both cerebral and extracerebral layers—represents a broad spectrum of noise sources and poses a significant challenge in signal analysis (Scholkmann et al., 2014, 2022; Tachtsidis and Scholkmann, 2016). Although considerable efforts have been invested in the development and validation of various correction methods to reduce different types of artifacts in fNIRS signals (e.g., Scholkmann et al., 2010; Cooper et al., 2012; Brigadoi et al., 2014; Pollonini et al., 2014, 2016; Tachtsidis and Scholkmann, 2016; Di Lorenzo et al., 2019; Novi et al., 2020b; Santosa et al., 2020; Wyser et al., 2020, 2022; Klein et al., 2022b), it is not necessarily possible to easily translate these algorithms for real-time use (cf. Figure 3). Real-time analysis comes with its own challenges, such as the need to preprocess data within a specific time frame to keep up with ongoing data collection (Lührs and Goebel, 2017). Additionally, some of these offline algorithms require parameter tuning, which may not be possible in real-time scenarios due to the limited data available.

### 3.1 Channel quality assessment and channel pruning

#### 3.1.1 Potential challenges

fNIRS is an optical imaging method whose signal quality depends largely on the direct contact of the optodes with the individual's skin. This so-called optode-scalp coupling can influence the signal-to-noise ratio of the measurement data. Poor or inconsistent contact can introduce noise and artifacts and affect data accuracy and reliability (Pollonini et al., 2014, 2016). In addition, the quality of fNIRS signals can be affected both by melanin in the skin, which reduces NIR light penetration, and by hair characteristics such as color, thickness and density, which absorb the light and reduce the signal quality (Pollonini et al., 2016; Yücel et al., 2021; Kwasa et al., 2023).

In offline analysis, it is common to perform signal quality assessment early in the preprocessing pipeline. This approach typically results in channels with poor signal quality being excluded (Pollonini et al., 2014; Sappia et al., 2020; Yücel et al., 2021), so that these channels no longer have any influence on the data interpretation (Hocke et al., 2018). In real-time applications, poor channel quality could have an immediate and detrimental impact on data accuracy. Accordingly, implementing real-time channel quality assessment could provide significant benefits. Such an approach could monitor and account for potential channel quality degradation during real-time analysis caused by factors such as variations in optode-scalp coupling or varying external environmental conditions. This would allow the system to identify and potentially ignore poor quality channels, maintaining the integrity of the real-time intervention over time.

#### 3.1.2 Possible options

Various metrics are available for (offline) assessment of the quality of fNIRS signals. A commonly used measure is the relative coefficient of variation (CV), which is calculated from raw light intensity data.

**Coefficient of variation (CV)**

The CV is a measure of the variability of the signal and is often expressed as a percentage in fNIRS research (Schmitz et al., 2005; Piper et al., 2014; Hocke et al., 2018)

$$\begin{array}{l} CV=\frac{\sigma_{\lambda }}{\mu_{\lambda }}\cdot 100\% \end{array}$$

where σ_λ_ represents the standard deviation and μ_λ_ the mean of the signal of a wavelength λ (Schmitz et al., 2005; Piper et al., 2014).

Calculating CV requires the use of raw light intensity data, as conversion to optical density values would result in mean values close to zero, causing the standard deviation to exceed the mean, resulting in an exaggerated, uninterpretable CV (Hocke et al., 2018). A higher CV indicates a lower signal-to-noise ratio, and channels with CVs exceeding a predefined threshold (e.g., CV = 15%; Piper et al., 2014; or CV = 7.5%; Hocke et al., 2018; Zimeo Morais et al., 2018) are typically excluded from subsequent preprocessing. However, it should be noted that these CV thresholds are usually chosen subjectively, which increases the risk of data manipulation and highlights the need for transparency and rigorous justification when setting these values. There is therefore an urgent need to determine threshold values empirically, using data-driven methods or based on theoretical frameworks. Besides, the CV may not only detect poor channel quality but also be sensitive to MAs (Hocke et al., 2018), which can lead to increased standard deviations in the signal. Finally, CV is a rather liberal metric, as channels with generally higher noise levels may not be reliably identified as poor quality channels.

Another quality metric is the signal quality index (SQI). This metric provides a quantitative assessment of the quality of the fNIRS signal, ranging from very low to very high quality (Sappia et al., 2020).

**Signal Quality Index (SQI)**

The SQI includes three different assessment stages that allow the quality of fNIRS signals to be evaluated. In the initial stage, very low quality signals are identified using three different features and their associated threshold values. The second stage then identifies very high quality signals based on a single feature and the corresponding threshold. In the third stage, the signals are finally rated on a scale of 1 to 5, which indicates the signal quality from very low to very high (Sappia et al., 2020).

By taking into account various features and their respective thresholds, the SQI provides a differentiated and comprehensive assessment of signal quality. However, it is important to note that the thresholds used in SQI to assess signal quality are based on heuristics derived from a single, relatively small data set (Sappia et al., 2020). Therefore, the generalizability of this quality metric to other data sets and experimental conditions requires further validation.

A more direct assessment of the quality of the optode-scalp coupling is done by calculating the scalp coupling index (SCI) (Pollonini et al., 2014, 2016).

**Scalp Coupling Index (SCI)**

The SCI is a metric for assessing the strength of cardiac oscillations within the raw light intensity (or optical density) data. The calculation is done by determining the zero-lag cross-correlation (*) of the signals at two wavelengths, λ_1_ and λ_2_ from the same channel (*y*_λ_1__ and *y*_λ_2__), which is finally normalized by the standard deviations of the respective signals σ_λ_1__ and σ_λ_2__ (Pollonini et al., 2014, 2016):

$$\begin{array}{l} SCI=\frac{y_{\lambda_{1}}}{\sigma_{\lambda_{1}}}*\frac{y_{\lambda_{2}}}{\sigma_{\lambda_{2}}} \end{array}$$

To calculate the SCI, the raw fNIRS signal is band-pass filtered to extract the cardiac signal component. Therefore, it is important to ensure that the sampling rate is at least twice the higher cutoff frequency to satisfy the Shannon-Nyquist theorem and thus avoid aliasing, which could distort the signal representation (Oshana, 2006; Pollonini et al., 2016). If a channel has an SCI value of 1, it means perfect signal quality and a threshold (e.g., *thresh* = 0.8) is usually set to classify channels as either good or poor quality. However, the SCI is also susceptible to the influence of MAs, which can contribute to falsely inflating the SCI value (Pollonini et al., 2016). To address this issue, the peak spectral power (PSP) metric was introduced as a complement to the SCI. The PSP quantifies the spectral power of the cross-correlated signal and an empirically determined threshold of PSP = 0.1 is used to further evaluate the signal quality (Pollonini et al., 2016). The SCI is a simple measure that takes physiological information into account and is well suited for assessing signal quality. However, its conservative nature may lead to the exclusion of potentially useful channels (Hocke et al., 2018).

#### 3.1.3 Further considerations

**Figure 4:**
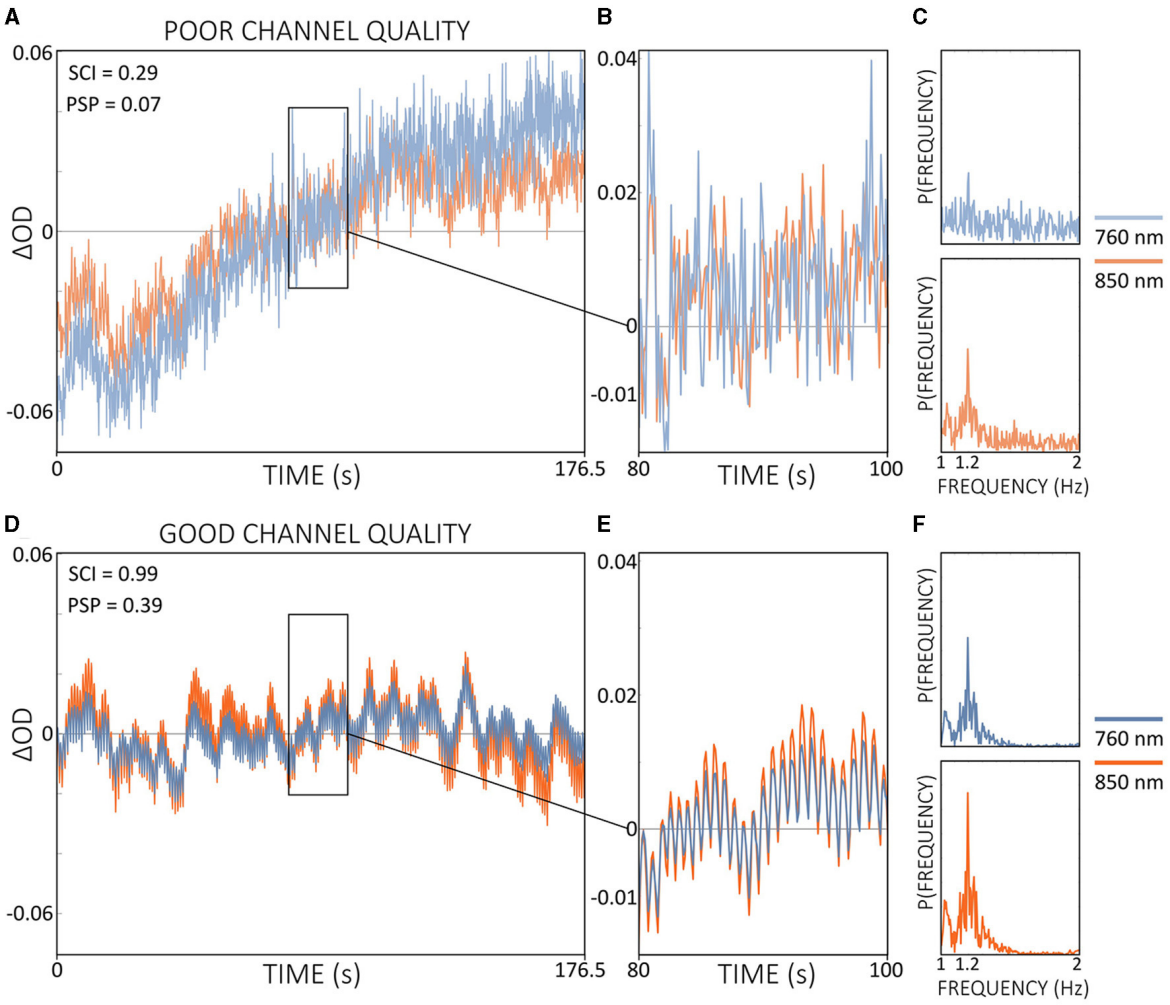
Comparison of signal qualities of a poor **(A)** and a good **(D)** channel of Δ*OD* at 760 and 850 nm during resting state data from a single subject. Enlarged signal windows in **(B, E)** illustrate the presence or absence of a clear cardiac pulsation. The corresponding power spectra resulting from a single-channel fast Fourier transform is shown in **(C, F)**. Quality metrics SCI and PSP are shown in **(A, D)**. Note that Δ*OD* data is visualized but SCI and PSP are calculated based on the raw light intensity data.

Current practice in real-time fNIRS research typically does not include continuous monitoring of channel quality during measurements (Kohl et al., 2020). In most fNIRS experiments, channel quality is generally assessed during the calibration phase, which occurs immediately before the actual experiment begins. Metrics such as CV are commonly used for this purpose and are built into some acquisition software. However, these metrics often lack crucial physiological information, such as cardiac pulsations, and therefore may not be the most appropriate metrics for assessing channel quality. As illustrated in Figure 4, SCI and the combination of SCI and PSP could accurately distinguish between good and poor channel quality, underlining the importance of including physiological information.

Integrating real-time quality control throughout the entire data acquisition process offers potential benefits for online fNIRS applications. For instance, the inclusion of channels with poor quality or those that experience sudden drops or degradation in quality during ongoing measurement could be avoided. The signal quality assessment tool *Placing Headgear Optodes Efficiently Before Experiment* (PHOEBE; https://github.com/lpollonini/phoebe) is a toolbox that already provides real-time quality control, integrating SCI and PSP (Pollonini et al., 2016). Although PHOEBE is actually designed for signal quality assessment before data collection begins, its functionality could be adapted for real-time fNIRS applications and potentially improve the reliability and efficiency of online experiments.

### 3.2 The modified Beer Lambert Law

#### 3.2.1 Potential challenges

A limitation of the widely used continuous-wave (CW) fNIRS systems is the inherent inability to directly derive absolute hemoglobin values. This limitation is due to the lack of a direct measurement of the optical properties of the underlying tissue (Scholkmann et al., 2014; Pinti et al., 2020). However, through certain reasonable assumptions, it is possible to estimate changes in hemoglobin concentration (Δ[*HbX*], including Δ[*HbO*] and Δ[*HbR*]) (Scholkmann et al., 2014; Pinti et al., 2020). These conversions are applied in both offline and real-time analysis. However, to ensure accurate conversions in real time, there are some important considerations to keep in mind.

#### 3.2.2 Possible options

In fNIRS, the modified Beer-Lambert law (mBLL) (Delpy et al., 1988) is fundamental for quantifying the interaction of light with biological tissue.

**modified Beer Lambert law (mBLL)**

The mBLL extends the traditional Beer Lambert law with a scattering-dependent light intensity loss parameter to account for scattering in biological tissue. The mBLL characterizes how light intensity decreases as it passes through tissue. In this context, the optical density (OD) is calculated as a dimensionless unit. OD represents the attenuation of light as it penetrates tissue. The mBLL relates the OD to the factors chromophore concentrations [*HbX*], the molar extinction coefficient (ϵ), the differential path length factor (DPF), the source-detector distance (*d*) and the scattering parameter *G* (Scholkmann et al., 2014):

$$\begin{array}{l} \begin{array}{c} OD\left(t,\lambda \right)=-\log\frac{I}{I_{0}}\\ =\underset{X}{\sum }\epsilon_{X}\left(\lambda \right)\left[HbX\right]\left(t\right)\cdot DPF\left(\lambda \right)\cdot d+G\left(\lambda \right) \end{array} \end{array}$$

Here, log denotes the logarithm with base 10 (decadic logarithm), and λ represents the wavelength and [*HbX*](*t*) represents the concentration of chromophore X at time *t*. In practice, *G* is often considered time invariant and negligible for calculating temporal changes in chromophore concentration when scattering changes are minimal compared to absorption. This simplification focuses the mBLL on absorption effects (Scholkmann et al., 2014):

(1) $$\begin{array}{l} \begin{array}{c} \Delta OD\left(\Delta t,\lambda \right)=-\log\frac{I\left(t_{1},\lambda \right)}{I\left(t_{0},\lambda \right)}\\ =\underset{X}{\sum }\epsilon_{X}\left(\lambda \right)\Delta \left[HbX\right]\cdot DPF\left(\lambda \right)\cdot d \end{array} \end{array}$$

Equation 1 reflects the change in optical density (Δ*OD*) between two time points, *t*_1_ and *t*_0_, with respect to the change in chromophore concentrations (Δ[*HbX*]). The scattering parameter *G* is omitted assuming that the scattering changes are small compared to the absorption changes (Scholkmann et al., 2014). Finally, assuming two wavelengths λ_1_ and λ_2_, the concentration changes of Δ[*HbO*] and Δ[*HbR*] can be calculated by rearranging the Equation (1) as follows:

$$\begin{array}{l} \left[\begin{array}{c} \Delta \left[HbO\right]\\ \Delta \left[HbR\right] \end{array}\right]=\frac{1}{d}{\left[\begin{array}{cc} \epsilon_{HbO,\lambda_{1}} & \epsilon_{HbR,\lambda_{1}}\\ \epsilon_{HbO,\lambda_{2}} & \epsilon_{HbR,\lambda_{2}} \end{array}\right]}^{-1}\left[\begin{array}{c} \frac{\Delta OD\left(\Delta t,\lambda_{1}\right)}{DPF\left(\lambda_{1}\right)}\\ \frac{\Delta OD\left(\Delta t,\lambda_{2}\right)}{DPF\left(\lambda_{2}\right)} \end{array}\right] \end{array}$$

The resulting unit is the molar concentration (M) and is often expressed in the range of μ*M* (Yücel et al., 2021).

The values of DPF and ϵ depend on the wavelength of the light used and the corresponding values are usually adopted from the existing literature (Duncan et al., 1995; Matcher et al., 1995; Jacques, 2013; Scholkmann et al., 2014). It is assumed that these empirically determined parameters remain constant (Scholkmann et al., 2014). The DPF, which acts as a correction factor, takes into account the effects of scattering in tissue and indicates the average path that photons travel from a source to a detector (Whiteman et al., 2017). It provides a single average value and neglects possible variations in light penetration depth at different distances between source and detector. For instance, with a source-detector distance of about 3 cm and an effective photon path length of about 18 cm (taking back and forth scattering into account), the corresponding DPF would be 6 (i.e., 6 × 3 cm = 18 cm) (Whiteman et al., 2017). This assumes a uniform and invariant spatial sensitivity profile across all source-detector pairs, which may not accurately capture the true depth of light penetration for a given channel. Furthermore, it has been shown that these parameters can also vary significantly during the experiment and that this variation can also be subject-dependent. These fluctuations are often overlooked and not taken into account in analyses (Zohdi et al., 2018). Moreover, it has been demonstrated that the DPF can be influenced by several other individual factors, including age and anatomical brain region (Scholkmann and Wolf, 2013; Whiteman et al., 2017).

#### 3.2.3 Further considerations

The mBLL plays a crucial role in detecting (real-time) changes in hemoglobin concentration based on CW-fNIRS data (Scholkmann et al., 2014; Pinti et al., 2020). For real-time conversions, it is important to ensure that a sufficiently long baseline (*I*_0_) has been recorded before Δ*OD* is calculated. While offline analyses typically use the entire recording as a baseline, real-time applications rely on a shorter, predefined baseline that is acquired before the actual real-time application starts. It is therefore important to ensure that the baseline is sufficiently long (Lührs and Goebel, 2017) and (mostly) free of artifacts. While a baseline as long as possible would be beneficial, implementation may not always be feasible, particularly in populations that tend to be more impatient, such as children. As a rough estimate, 20–30 s of largely artifact-free data can be considered a starting point for real-time applications. Furthermore, considering age-dependent correction of the DPF could be a promising strategy to improve real-time processing. However, research is needed in both contexts to evaluate their potential effectiveness in improving the accuracy of CW-fNIRS data processing in real-time scenarios.

### 3.3 Motion artifact correction

#### 3.3.1 Potential challenges

**Figure 5:**
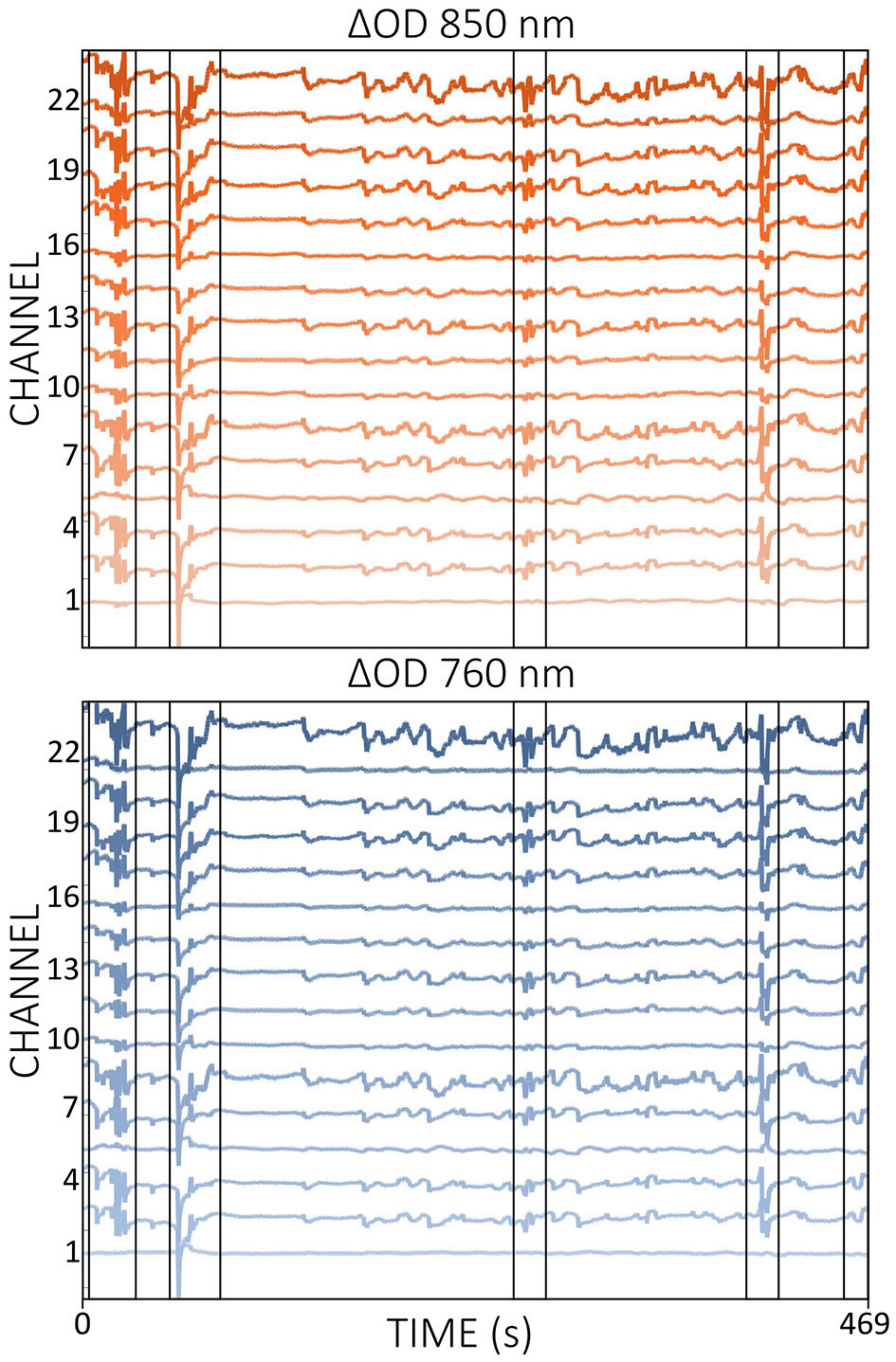
Illustration of MAs (some are black framed) in Δ*OD* data for wavelengths 760 nm (blue) and 850 nm (red). The data displayed is resting-state data from a single subject.

A frequently mentioned advantage of fNIRS compared to other brain imaging methods is its tolerance to (head) motion (Scarapicchia et al., 2017; Fishburn et al., 2019; Pinti et al., 2020; Huang et al., 2022). However, MAs can also present a challenge for fNIRS data, particularly in populations that are difficult to instruct to keep their heads still, such as toddlers and infants (Di Lorenzo et al., 2019; Fishburn et al., 2019; Gemignani and Gervain, 2021; Yücel et al., 2021). As shown in Figure 5, MAs often appear as abrupt signal changes, such as spikes, slow drifts and baseline shifts, which are mainly due to optode and/or cap movements (Di Lorenzo et al., 2019; Von Lühmann et al., 2019; Gemignani and Gervain, 2021), but also more subtle movements such as jaw movements that trigger activity in the temporalis muscle can contribute to MAs (Zimeo Morais et al., 2017). The resulting changes in the shape of the fNIRS signal can alter the shape of the underlying hemodynamic response function (HRF), thereby complicating interpretation (Brigadoi et al., 2014). The occurrence of MAs is particularly critical in real-time applications, as abrupt signal changes and spikes can have significant effects on the feedback. In theory, most MAs should be relatively straightforward to correct because they often manifest as rapid changes within a slow hemodynamic signal. In practice, however, the effectiveness of the correction process can depend heavily on the specific method used and not all existing (offline) algorithms can be easily adapted for use in real time.

#### 3.3.2 Possible options

There are several validated MA correction methods for offline analysis (Zhang et al., 2005; Cui et al., 2010; Scholkmann et al., 2010; Molavi and Dumont, 2012; Yücel et al., 2014; Delgado Reyes et al., 2018; Jahani et al., 2018; Di Lorenzo et al., 2019; Fishburn et al., 2019; Von Lühmann et al., 2019; Novi et al., 2020a; Huang et al., 2022). However, some of these methods require an initial motion detection step that involves parameter tuning based on specific thresholds (Di Lorenzo et al., 2019; Fishburn et al., 2019). While parameter tuning can be beneficial for targeting specific affected parts of the signal, it can be challenging due to participant and instrument variability (Fishburn et al., 2019). Consequently, these methods are rather impractical for real-time applications. However, there are several alternative MA correction methods that are suitable for real-time analysis because they work automatically without the need for parameter tuning or prior artifact detection steps.

One such method is the correlation-based signal improvement (CBSI) approach (Cui et al., 2010).

**Correlation-based Signal Improvement (CBSI)**

According to Cui et al. (2010), the noisy Δ[*HbO*] (*y*_*HbO*_*MA*__) and Δ[*HbR*] signals (*y*_*HbR*_*MA*__) affected by MAs can be represented as:

$$\begin{array}{l} \begin{array}{c} y_{HbO_{MA}}=y_{HbO_{ideal}}+\alpha MA\\ y_{HbR_{MA}}=y_{HbR_{ideal}}+MA \end{array} \end{array}$$

where *y*_*HbO*_*ideal*__ and *y*_*HbR*_*ideal*__ represent the ideal (i.e., without MAs) Δ[*HbO*] and Δ[*HbR*] signals, respectively. The MA has the same effect on Δ[*HbO*] and Δ[*HbR*] which is subject to a positive factor α and is defined as the ratio of the noise amplitude in Δ[*HbO*] and Δ[*HbR*]. The CBSI method is based on two assumptions. First, a perfect negative (i.e., correlation = −1) relationship between *y*_*HbO*_*ideal*__ and *y*_*HbR*_*ideal*__ is assumed (Cui et al., 2010):

$$\begin{array}{l} y_{HbO_{ideal}}=-\beta y_{HbR_{ideal}} \end{array}$$

where β is defined as the ratio of the amplitude in the noise-free Δ[*HbO*] and Δ[*HbR*] (Cui et al., 2010). Second, *y*_*HbO*_*ideal*__ and the MAs are assumed to be uncorrelated (i.e., correlation = 0):

(2) $$\begin{array}{l} \begin{array}{c} y_{HbO_{ideal}}=\frac{1}{2}\left(y_{HbO_{MA}}-\alpha y_{HbR_{MA}}\right)\\ y_{HbR_{ideal}}=-\frac{1}{\alpha }y_{HbO_{ideal}} \end{array} \end{array}$$

Assuming that α = β, it was shown that the parameter α is calculated as the quotient of the standard deviations of *y*_*HbO*_*MA*__ and *y*_*HbR*_*MA*__ (for more details see Cui et al., 2010). With this result, the corrected signals *y*_*HbO*_*ideal*__ and *y*_*HbR*_*ideal*__ are finally estimated (cf. Equation 2) (Cui et al., 2010).

The CBSI method has been proposed as a suitable automated MA correction method for real-time preprocessing in NFB applications (Kohl et al., 2020), but its performance in real time has not yet been validated. However, some criticisms of the CBSI method have also been raised. One of the main concerns is that it relies on correlation assumptions that are often not met in real data sets. Furthermore, the CBSI method alters the Δ[*HbR*] signal in a way that differs from its original representation (cf. Equation 2 and Figure 7C) (Brigadoi et al., 2014; Di Lorenzo et al., 2019; Fishburn et al., 2019; Novi et al., 2020b). In addition, changing Δ[*HbR*] may affect other derived variables, such as total hemoglobin (Δ[*HbT*] = Δ[*HbO*] + Δ[*HbR*]) and hemoglobin differences (Δ[*HbDiff*]). = Δ[*HbO*] - Δ[*HbR*]), two variables that have been also analyzed in fNIRS research (Lu et al., 2015; Hakim et al., 2022). Accordingly, the CBSI method may not be the preferred option for (real-time) MA correction.

The temporal derivative distribution repair (TDDR) method introduced by Fishburn et al. (2019) is also a fully automated MA correction algorithm. This method is based on three assumptions related to the first (i.e., temporal) derivative of the fNIRS signals: (1) the measured activation is unrelated to MAs and is approximately normally distributed, (2) the majority of the signal is free of MAs, and (3) MAs have a much larger amplitude compared to artifact-free parts of the signal. By using the temporal derivative, which essentially represents the signal changes over time, the TDDR method effectively identifies MAs as outliers in the signal (Fishburn et al., 2019).

**Temporal Derivative Distribution Repair (TDDR)**

The TDDR method uses an iterative reweighting scheme similar to robust regression. To do this, the TDDR algorithm calculates the time derivative of the signal for each sample, given by ẏ, and initializes the observation weights *w* to 1. The robust observation weights are then estimated iteratively until convergence (Fishburn et al., 2019). The estimation process begins by calculating the weighted average of the signal fluctuations, represented as

$$\begin{array}{l} \mu \left(t\right)=\frac{1}{\sum w}\sum wẏ\left(t\right) \end{array}$$

Next, the robust standard deviation of the residuals σ(*t*) is computed by finding the median of the absolute differences between the temporal derivative and the weighted mean. A scaling factor *k* is applied to ensure compatibility with normally distributed data. Scaled deviations, *d*(*t*), are calculated by dividing the residuals ϵ(*t*) by the product of σ(*t*) and an optimization parameter *c* = 4.685 (Fishburn et al., 2019). New weights, *w*(*t*), are then determined based on *d*(*t*) using Tukey's biweight function, which is known to be robust to outliers:

$$\begin{array}{l} w\left(t\right)=\{\begin{array}{ll} {\left(1-d{\left(t\right)}^{2}\right)}^{2}, & \text{if }d\left(t\right)<1.\\ 0, & \text{otherwise}. \end{array} \end{array}$$

The iterative process continues until convergence is achieved, indicated by the stabilization of μ(*t*). Finally, the corrected signal *y*_*c*_(*t*) is obtained by integrating the weighted and centered signal *y* using the computed weights.

$$\begin{array}{l} y_{c}\left(t\right)=\overset{N}{\underset{t=1}{\sum }}w\left(t\right)\left(y\left(t\right)-\mu \left(t\right)\right) \end{array}$$

Since the calculations are based on the first derivative of the signal, the application of the algorithm depends on the sampling frequency, because the sampling frequency and the magnitude of the first derivative are directly related. To account for this issue, the data is separated into low and high frequency components using a low-pass filter with a cut-off frequency of *f*_*low*_ = 0.5 Hz (Fishburn et al., 2019). Then TDDR is applied only to the low-frequency part of the derivative and after correction, the high-frequency parts are integrated back into the signal (Fishburn et al., 2019). This limitation is probably acceptable because subsequent processing steps often use additional band-pass filter steps with lower cut-offs to reduce high-frequency components in the signal (Pinti et al., 2019). Another limitation of TDDR, shared with many other MA correction methods, is that it primarily corrects MAs such as spikes and baseline shifts, while MAs with low amplitudes or slow drifts may remain uncorrected (Fishburn et al., 2019). However, TDDR offers the advantage of simple implementation and fast processing, which makes it particularly interesting for real-time application.

**Figure 6:**
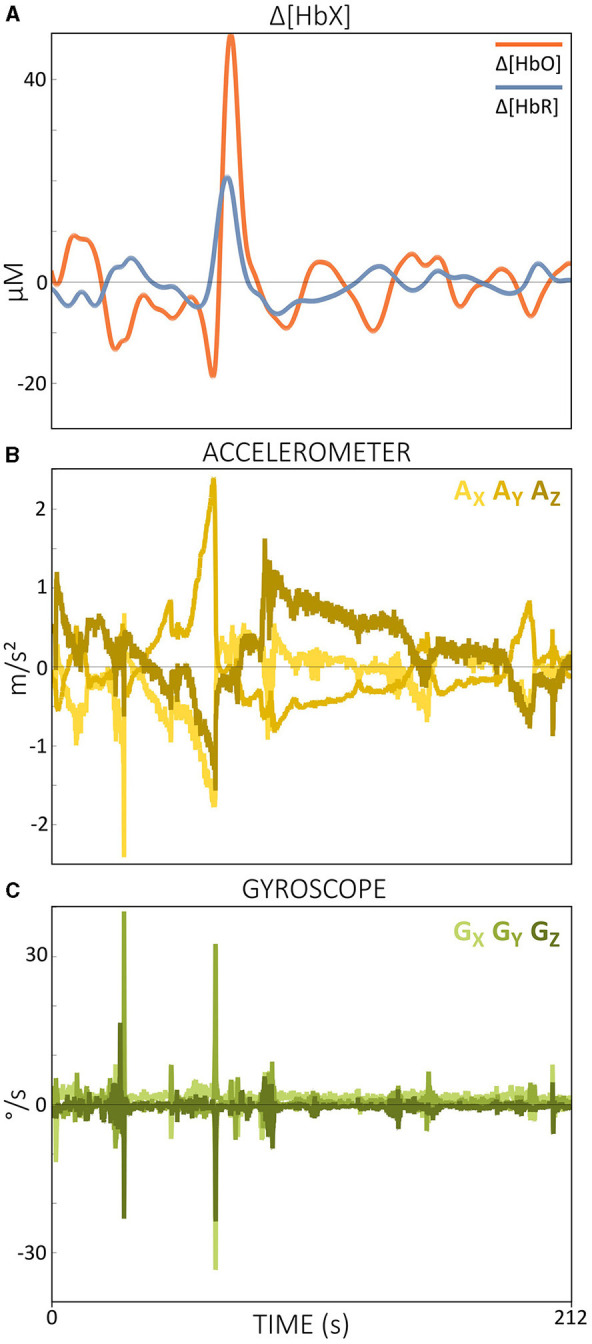
Visualization of **(A)** Δ[*HbX*] signals including MAs, **(B)** corresponding 3D accelerometer data and **(C)** 3D gyroscope data. The data displayed is motor execution data from a single subject.

Another possible option for real-time MA correction is to utilize information from an inertial measurement unit (IMU) that collects auxiliary information such as accelerometer, gyroscope, and magnetometer data directly at the optode level (Virtanen et al., 2011; Metz et al., 2015; Siddiquee et al., 2018; Von Lühmann et al., 2019; von Lühmann et al., 2020a). Such a correction method uses the time-stamped head motion information (Figure 6) and corrects MAs in the fNIRS signal through a regression-based approach, for instance, in the context of a general linear model (GLM) (von Lühmann et al., 2020a).

**GLM-filter using Inertial Measurement Units (IMUs)**

GLMs are widely used in both fMRI and fNIRS analysis to estimate brain activation associated with a task being performed (Huppert, 2016; von Lühmann et al., 2020a). In fNIRS, the measured data *Y* is typically modeled using a design matrix *X*_*task*_ which consists of task-related regressors. These regressors represent the temporal structure of the experiment with respect to task onsets and are usually derived by integrating an a-priori hypothesis about the underlying HRF via a basis function that characterizes the expected shape (Huppert, 2016; von Lühmann et al., 2020a):

$$\begin{array}{l} Y=X_{task}\cdot \beta_{task}+\epsilon \end{array}$$

Here, β_*task*_ is a vector indicating the strength of the relationship between the signals in *Y* and the design matrix *X*_*task*_, while the residual term ϵ represents the error or part of the data that cannot be explained by the model. To account for MAs in the signal *Y*, the IMU *X*_*IMU*_ data can be added to the design matrix of the GLM:

$$\begin{array}{l} Y=\left[\begin{array}{cc} X_{task} & X_{IMU} \end{array}\right]\cdot \left[\begin{array}{c} \beta_{task}\\ \beta_{IMU} \end{array}\right]+\epsilon \end{array}$$

where β_*IMU*_ quantifies the contribution of MAs to the signal *Y*. This approach has already been used in fNIRS studies to improve the estimation of task-related activation (von Lühmann et al., 2020a). For real-time applications that require a cleaned time-series signal, a special GLM filter can be applied that includes only the IMU data as regressors:

(3) $$\begin{array}{l} Y=X_{IMU}\cdot \beta_{IMU}+\epsilon \end{array}$$

In this approach, ϵ is assumed to represent the cleaned fNIRS data *Y*_*clean*_, which can be calculated by rearranging the Equation (3) to

$$\begin{array}{l} \begin{array}{c} \epsilon =Y-X_{IMU}\cdot \beta_{IMU}\\ =Y_{clean} \end{array} \end{array}$$

Although this simple implementation requires additional hardware, it is a promising option for MA correction in real-time applications. However, one of the challenges with this method is managing the synchronization between the occurrence of MAs in auxiliary signals and fNIRS data (cf. Figure 6) (von Lühmann et al., 2020a). To address this issue, von Lühmann et al. (2020a) proposed a possible solution by combining the (offline) GLM with the temporally embedded canonical correlation analysis (tCCA). tCCA is an extension of CCA, a dimensionality reduction technique for correlation analysis that finds linear combinations between two sets of time series while maximizing their correlation (Zhuang et al., 2020). In particular, (t)CCA reduces the risk of overfitting and shortens computation time by setting a correlation threshold above which components are considered artifacts. tCCA additionally introduces time-shifted versions of the nuisance signals and can therefore be used to effectively detect and correct MAs in the fNIRS data (von Lühmann et al., 2020a; Zhuang et al., 2020). To optimize accuracy, the method relies on key parameters such as step size (τ_*D*_), maximum time delay (Δ*t*), and a correlation threshold (ρ_*thresh*_). Globally optimized values have been suggested, but individual parameter tuning might further improve performance (von Lühmann et al., 2020a).

#### 3.3.3 Further considerations

Real-time MA correction is not yet often used in fNIRS, as evidenced by the limited number of studies that have reported its use in NFB studies (Kohl et al., 2020). However, addressing MAs in real time is critical to ensure good signal quality and provide accurate and reliable feedback to the user during online experiments. A regression-based approach using IMUs could be a promising solution for real-time MA correction since MAs are directly taken into account at the optode level. Since the use of tCCA led to a significant improvement in MA correction for offline analyzes (von Lühmann et al., 2020a), this could also be interesting for real-time applications. Correcting MAs using IMUs by approximating the time lag between them and the slower hemodynamic response is a practical and promising real-time approach. However, despite the promising potential in offline validations (von Lühmann et al., 2020a), implementation in real-time fNIRS applications has not yet been demonstrated.

**Figure 7:**
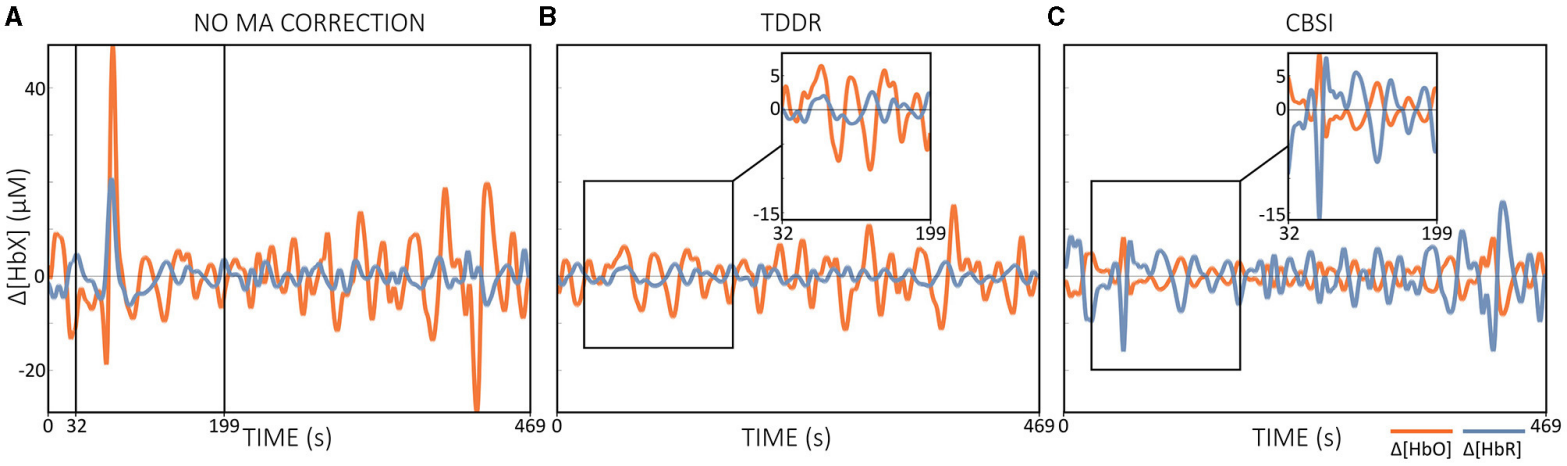
Illustration of the effect of offline MA correction. **(A)** shows only band-pass filtered Δ[*HbX*] data without MA correction, **(B)** Δ[*HbX*] data corrected with TDDR + band-pass filtering and **(C)** Δ[*HbX*] data corrected with CBSI + band-pass filtering. Applied filter was a zero-phase 2nd order Butterworth filter with cut-off frequencies of [0.01, 0.09] Hz and all preprocessing steps were performed offline. The data displayed is motor execution data from a single subject.

If IMU hardware is not available, the TDDR method could be a promising option for online MA correction. The TDDR method offers several advantages, including automatic operation without the need for parameter tuning, ease of implementation, and fast convergence (Fishburn et al., 2019). Compared to the CBSI method, which removes MAs but changes the Δ[*HbR*] signal to become a modified version of Δ[*HbO*] (cf. Figure 7A vs. Figure 7C), the TDDR method effectively removes MAs without profound signal changes (cf. Figure 7A vs. Figure 7B). Based on the general results of several studies investigating offline MA correction techniques, due to the dependence of the CBSI method on strong assumptions, its application for both offline and real-time applications should be carefully considered (Brigadoi et al., 2014; Di Lorenzo et al., 2019; Fishburn et al., 2019; Novi et al., 2020b). The question of the optimal method for real-time MA correction cannot currently be answered conclusively and requires further investigation. Future research is needed to compare and evaluate potential MA correction techniques for use in fNIRS-based real-time applications and to determine the most effective and reliable approach to improving the quality and accuracy of real-time fNIRS data.

### 3.4 Temporal filtering

#### 3.4.1 Potential challenges

A large portion of the recorded fNIRS signal contains non-evoked extracerebral and cerebral systemic artifacts. These artifacts originate from spontaneous physiological changes such as heartbeat (~1 Hz), respiration (~0.3 Hz), Mayer waves (~0.1 Hz), and very low-frequency oscillations (~0.01–0.05 Hz) (Scholkmann et al., 2014; Wyser et al., 2020). Since some of these artifact frequencies have only little overlap with the typically used task frequencies (~0.02 Hz < *f*_*t*_ < 0.05 Hz), it is possible to reduce this physiological noise with conventional temporal filters (Pinti et al., 2019). The application of temporal filters for artifact reduction is common in both offline and real-time fNIRS analysis (Pinti et al., 2019; Kohl et al., 2020). However, not every offline filter can be applied directly in real-time. One of the reasons for this is that offline filters have access to the entire data set, allowing the implementation of more sophisticated and computationally intensive filtering techniques. However, in real-time scenarios, the available data window for filtering remains limited and is continuously updated with new data points, which is why real-time filtering is often based on the use of simpler and more computationally efficient algorithms (Lührs and Goebel, 2017).

Filtering is a fundamental aspect of digital signal processing that is explained in detail in various resources (e.g., Smith, 1997; Widmann et al., 2015; De Cheveigné and Nelken, 2019). Therefore, the following section covers only the most basic parts that could likely be relevant to designing a filter for fNIRS-based real-time analysis.

#### 3.4.2 Possible options

In general, a filter is a system that produces an output signal by applying certain weights to an input signal (De Cheveigné and Nelken, 2019).

**Digital Filter**

A digital filter works by convolving the input signal *y* (e.g., the fNIRS signal) with an impulse response (IR) (*h*(*n*), where *n* = 0, ..., *N*) at each time point *t* (De Cheveigné and Nelken, 2019). The output signal *y*_*filter*_(*t*) is obtained as the sum of the weighted contributions of *N* points of the input signal:

$$\begin{array}{l} y_{filter}\left(t\right)=\overset{N}{\underset{n=0}{\sum }}h\left(n\right)y\left(t-n\right) \end{array}$$

Accordingly, each point of the output signal *y*_*filter*_(*t*) is influenced by *N* points of the input signal *y*(*t*), which leads to a temporal smearing effect whose properties, such as signal smoothing, are determined by the IR function *h*(*n*) (De Cheveigné and Nelken, 2019).

**Impulse Response (IR) Functions**

The IR of the filter characterizes its behavior and can be divided into two main types: finite impulse response (FIR) filter and infinite impulse response (IIR) filter (Widmann et al., 2015; De Cheveigné and Nelken, 2019; Pinti et al., 2019). FIR and IIR filters differ in their phase response, stability, and filter order (De Cheveigné and Nelken, 2019; Pinti et al., 2019):

- Phase response describes how much the filter delays each frequency component of the input signal. FIR filters typically have linear phase responses, which means that they introduce a constant delay across all frequency components of the input signal. In contrast, IIR filters generally have nonlinear phase responses, resulting in frequency-dependent delays (Widmann et al., 2015; De Cheveigné and Nelken, 2019).
- Stability refers to the ability of a filter to produce a finite output based on a finite input. With a stable filter, uncontrolled oscillations do not occur and the amplitude does not grow to infinity. This is important for reliable signal processing and to avoid signal distortion. FIR filters are always stable. IIR filters, on the other hand, can be stable or unstable depending on their design (Widmann et al., 2015; De Cheveigné and Nelken, 2019; Pinti et al., 2019).
- The filter order represents the number of previous input samples used to calculate a single output sample. A higher filter order thus indicates that the filter relies on more past samples to determine the current output. For FIR filters, the filter order is determined by the number of coefficients used in the IR function. IIR filters typically have lower filter orders compared to FIR filters because their reliance on feedback loops allows them to achieve similar filtering effects with fewer coefficients, (Widmann et al., 2015; De Cheveigné and Nelken, 2019; Pinti et al., 2019).

IIR filters such as the Butterworth filter are widely used in offline and real-time preprocessing of fNIRS. The moving average filter, on the other hand, is an example of a FIR filter used in real time (Pinti et al., 2019; Kohl et al., 2020).

For task-related analysis in fNIRS, designing a filter that preserves the task frequency of interest (*f*_*t*_) is crucial (Pinti et al., 2019).

**Task Frequency**

The task frequency (*f*_*t*_) is defined as the inverse of the sum of the task period (*t*_*t*_) and the rest period (*t*_*r*_) (Pinti et al., 2019)

$$\begin{array}{l} f_{t}=\frac{1}{\left(t_{t}+t_{r}\right)} \end{array}$$

For instance, in a block design with a task and rest period of 15 s each, *f*_*t*_ would be ~0.03 Hz ($\frac{1}{30}$ Hz). If the rest period is jittered (i.e., different rest lengths occurring in a pseudo-randomized order) a task frequency range ([*f*_*t*_*low*__, *f*_*t*_*high*__]) should be considered (Pinti et al., 2019). However, to finally determine the cut-off frequencies for the filter, it should be determined which type of filter to use.

**Filter Type**

**Figure 8:**
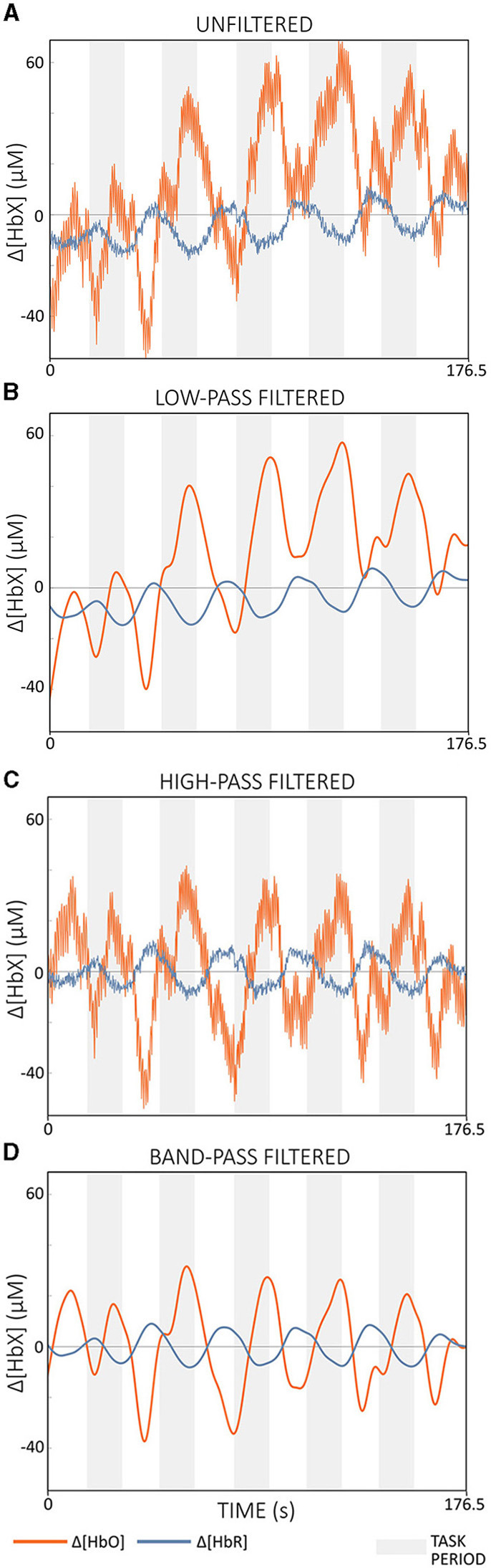
Illustration of the effects of different filter types applied to Δ[*HbO*] (red) and Δ[*HbR*] (blue) of semi-simulated data based on resting state data from a single subject. **(A)** shows the unfiltered signals. **(B)** Demonstrates the result of a low-pass filter with *f*_*t*_*low*__ = 0.09 Hz, effectively smoothing the signal. **(C)** shows the outcome of a high-pass filter with *f*_*t*_*high*__ = 0.01 Hz, effectively removing slow drifts. **(D)** shows the output of a band-pass filter with a cut-off frequency range of [0.01, 0.09] Hz. The filters were applied offline using a zero-phase 2nd order Butterworth filter. The gray areas indicate task periods.

Filter types commonly used in fNIRS research include high-pass, low-pass, and band-pass filters (Pinti et al., 2019; Kohl et al., 2020). High-pass filters attenuate frequencies below a certain cut-off frequency (*f*_*high*_), effectively eliminating slow drift and trends while centering the signal around zero (Figure 8A vs. Figure 8C). Low-pass filters, on the other hand, attenuate frequencies above a cut-off frequency (*f*_*low*_), resulting in a smoother output signal (Figure 8A vs. Figure 8B). Band-pass filters combine the effects of high- and low-pass filters, only allowing frequencies within a certain range ([*f*_*high*_, *f*_*low*_]) to pass (Figure 8A vs. Figure 8D) (Widmann et al., 2015; De Cheveigné and Nelken, 2019).

In addition to the filter type, a decision should be made as to whether a causal or acausal filter should be used.

**Causal vs. Acausal Filters**

**Figure 9:**
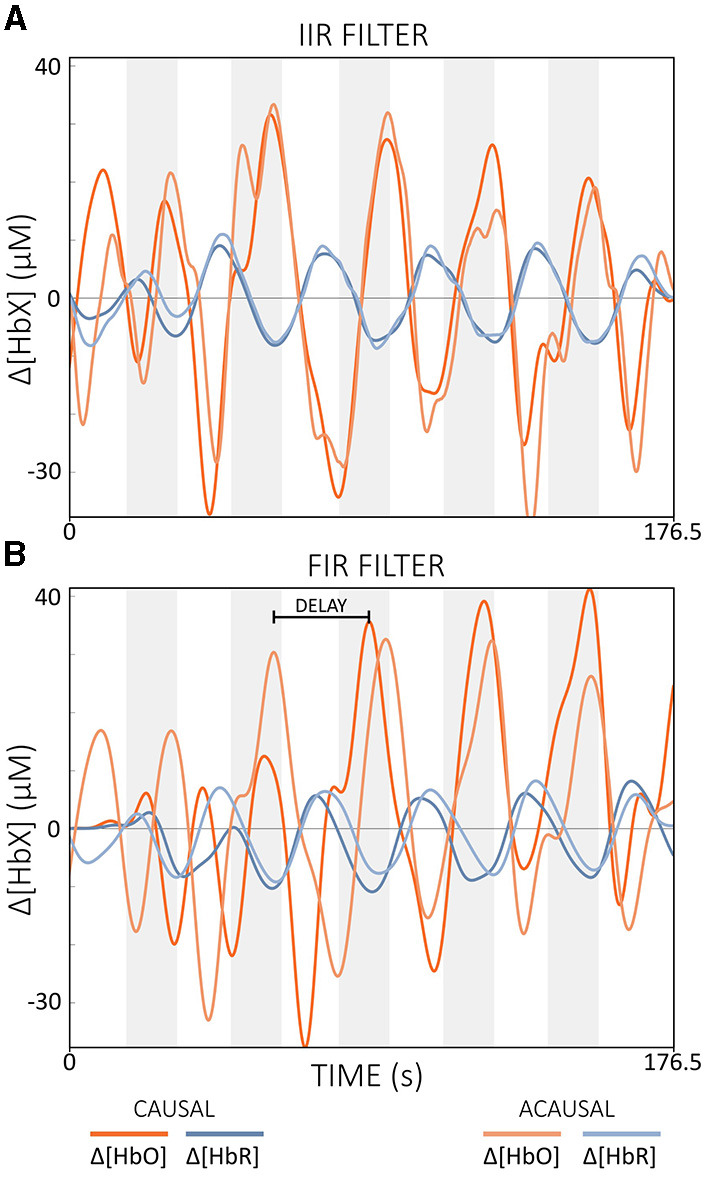
Illustration of the effect of causal and acausal filters for **(A)** IIR and **(B)** FIR filters. IIR: filter order = 2, Butterworth; FIR: filter order = 450. Note that band-pass filters with [0.01, 0.09] Hz were applied offline. The gray areas indicate task periods. The data displayed is semi-simulated data based on resting state data from a single subject.

Acausal filters include both past and future data points in their calculations. This allows them to achieve zero-phase properties and minimize phase distortion (Widmann et al., 2015; De Cheveigné and Nelken, 2019; Pinti et al., 2019). Causal IIR filters use only past and current data while achieving similar output to acausal IIR filters, which minimize phase distortion but are based on both past and future data (cf. Figure 9A). With FIR filters, causal versions lead to significantly larger phase delays due to the typical high filter order, while the acausal types can eliminate these delays through bidirectional processing (cf. Figure 9B) (Widmann et al., 2015).

Accordingly, causal filters, especially causal IIR filters, seem to be particularly well suited for real-time applications.

#### 3.4.3 Further considerations

**Figure 10:**
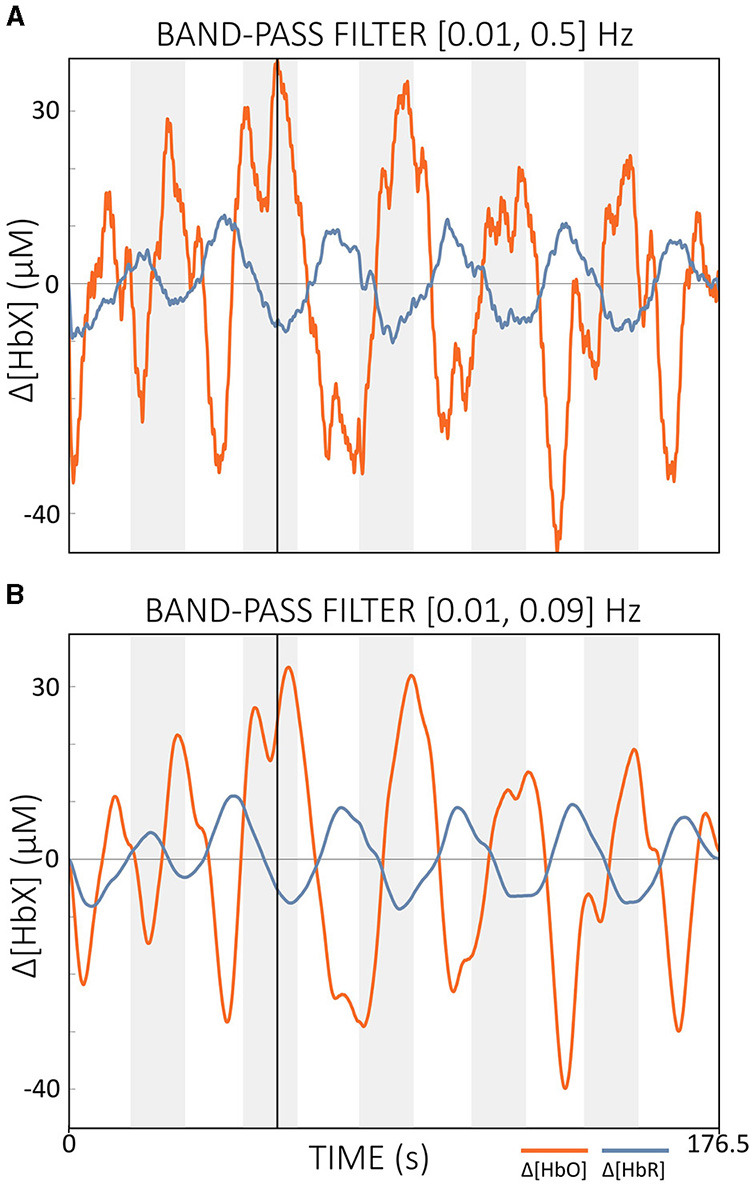
Comparison of offline filtered data with different cut-off frequencies. **(A)** Data filtered with cut-offs of [0.01, 0.5] Hz and **(B)** data filtered with cut-offs of [0.01, 0.09] Hz. The vertical black line indicates the same time point in both figures. The data displayed is semi-simulated data based on resting state data from a single subject.

According to Kohl et al. (2020), a filter step is often integrated into the real-time data processing pipeline of many fNIRS-guided NFB studies. Popular filter implementations include moving average (FIR) filters with lengths of 2–5 s and band-pass IIR filters with cut-off frequencies between 0.01 and 1.5 Hz (Kohl et al., 2020). For offline preprocessing, Pinti et al. (2019) proposed a zero-phase FIR band-pass filter with cut-off frequencies of [0.01, 0.09] Hz. This band effectively reduced physiological noise while preserving task-related and additional important information such as task frequency harmonics (Pinti et al., 2019). While FIR filters are efficient for offline processing, they can introduce significant delays when configured as causal filters for real-time applications, making them less suitable where low signal delay is critical. In contrast, IIR filters such as Butterworth filters, when implemented as causal filters, require less computational effort and typically have lower delays compared to causal FIR filters, although they still cannot reach the zero-delay performance of acausal filters. The resulting delay depends mainly on the cut-off frequencies chosen. Higher cutoff frequencies (above 0.1 Hz) can reduce delay but retain more physiological noise, potentially affecting real-time performance (cf. Figure 10A). The cut-off frequencies recommended by Pinti et al. (2019) are therefore probably also suitable for real-time applications, as long as they are not too close to the task frequency, as the resulting delay appears manageable (cf. Figures 10A, B).

### 3.5 Extracerebral systemic activity correction

#### 3.5.1 Potential challenges

Another source of noise in fNIRS is the extracerebral systemic artifacts, which have both non-evoked and task-evoked components (Scholkmann et al., 2014; Tachtsidis and Scholkmann, 2016). These artifacts arise from changes in blood flow and oxygen supply in the extracerebral layers (i.e., scalp, skull and cerebrospinal fluid) which are influenced by systemic physiological fluctuations, local conditions of the scalp and the subject's movements. The fact that NIR light penetrates these extracerebral tissues twice, once upon entry and once upon exit, makes the measured signals susceptible to contamination from these non-neural sources (Klein and Kranczioch, 2019). This type of noise can lead to possible misinterpretation of the fNIRS data. For instance, Caldwell et al. (2016) showed that systemic activity can either falsely mimic functional activation (false positives) or mask actual activation (false negatives) and highlighted that extracerebral and systemic factors significantly influence hemodynamic signals, sometimes triggering almost all of the recorded changes. In their study, they identified several sources of extracerebral contamination that can significantly influence the interpretation of neuronal activity from fNIRS data. These causes are mainly related to changes in blood flow and oxygen supply in the extracerebral layers, which are influenced by a variety of factors. These factors include autonomic nervous system activity, which in turn can be modulated by stress, emotions or even temperature fluctuations, as well as movements of the subject, which can lead to changes in scalp blood flow or a change in the optical properties of extracerebral tissue (Caldwell et al., 2016). Identifying and mitigating these confounding factors is therefore crucial for reliable data interpretation (Tachtsidis and Scholkmann, 2016). Of particular concern here are the task-related extracerebral components, as the frequency components of the artifact may overlap with the frequency of the task being performed, rendering traditional temporal filters ineffective for correction (Kirilina et al., 2012; Wyser et al., 2020; Klein et al., 2022b). However, recent hardware advances have produced a promising solution that allows direct correction of extracerebral systemic activity components and thus may help improve the accuracy and reliability of fNIRS signal analysis (Scholkmann et al., 2014; Tachtsidis and Scholkmann, 2016; Santosa et al., 2020; Wyser et al., 2020; Yücel et al., 2021; Abdalmalak et al., 2022; Klein et al., 2022b). Of course, correcting these artifacts is also critical for real-time applications, although this has not been done frequently to date (Kohl et al., 2020). Failure to correct for extracerebral systemic artifacts may result in misleading interpretations of brain activity, which could result in delays or inaccuracies in real-time applications, thereby negatively impacting user experience, performance and/or therapy outcomes.

#### 3.5.2 Possible options

**Figure 11:**
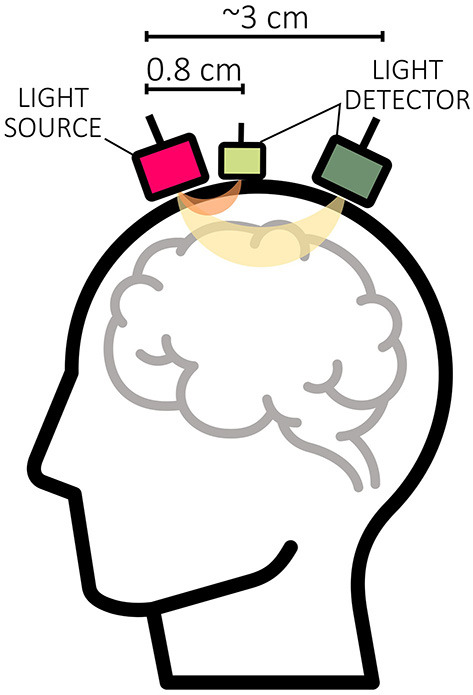
Illustration of the penetration depth of a short-distance channel (0.8 cm source-detector distance) as compared to a regular-distance channel (~3 cm source-detector distance).

Currently, short-distance channels (SDCs) are considered the gold standard for correcting extracerebral systemic activity, and several methods for integrating SDC signals are available that aim to improve the accuracy of regular-distance channels (RDC; ~3 cm source-detector distance) (Saager and Berger, 2005; Santosa et al., 2020; Von Lühmann et al., 2020b; Wyser et al., 2020, 2022; Abdalmalak et al., 2022; Klein et al., 2022b). An SDC is created by positioning a source and detector <10 mm apart, ideally 8.4 mm (in adults; Brigadoi and Cooper, 2015). Since the penetration depth of an fNIRS channel is about half the distance between source and detector, SDCs mainly detect the activity of extracerebral tissue (cf. Figure 11) (Santosa et al., 2020; Wyser et al., 2020; Klein et al., 2022b).

A simple regression-based correction method that uses SDCs is the short separation regression (SSR) approach (Saager and Berger, 2005; Klein et al., 2022b).

**Short-Separation Regression (SSR)**

The SSR method corrects for extracerebral systemic artifacts by subtracting the SDC signal multiplied by a scaling factor α from each RDC signal. This process yields the corrected signal (*y*_*clean*_) that represents the actual task-evoked brain activation:

(4) $$\begin{array}{l} y_{clean}=y_{RDC}-\alpha \cdot y_{SDC} \end{array}$$

The scaling factor α is determined by the quotient of two dot products (〈·, ·〉):

(5) $$\begin{array}{l} \alpha =\frac{\langle y_{SDC},y_{RDC}\rangle }{\langle y_{SDC},y_{SDC}\rangle } \end{array}$$

The dot product (〈*y*_*SDC*_, *y*_*RDC*_〉) quantifies the relationship between the SDC and RDC signals, while the dot product (〈*y*_*SDC*_, *y*_*SDC*_〉) represents the magnitude of the SDC signal. As a result, α quantifies the influence of the SDC signal (*y*_*SDC*_) on the RDC signal (*y*_*RDC*_) (Fang and Boas, 2009). It thus indicates the extent to which the SDC influences the RDC signal and provides a measure of how much correction is required to obtain a more accurate representation of the actual underlying brain activation.

There are theoretically different ways to define *y*_*SDC*_ in the Equations (4, 5). Possible options would be to use the SDC that is spatially closest to the RDC to be corrected, the SDC with the highest correlation to the RDCs, the average of all available SDCs, or the first *m* principal components (PCs) obtained from a principal component analysis (PCA) across all available SDCs. Due to its simplicity, the SSR method is well suited for real-time correction. However, when used without prior channel quality assessment, there is a risk that SDCs with poor signal quality will introduce noise into the RDC data (Klein et al., 2022b).

A more advanced regression-based method to correct for extracerebral systemic activity is to include the SDCs as (additional) regressors in a GLM framework (Santosa et al., 2020; von Lühmann et al., 2020a; Wyser et al., 2020; Klein et al., 2022b; Cockx et al., 2023).

**GLM filter using SDCs**

As shown in Equation (3) in Section 3.3, additionally recorded signals like IMUs can be used as regressors in the design matrix of a GLM. This also applies to SDCs, which can be used either alone or in combination with other nuisance regressors (Santosa et al., 2020; von Lühmann et al., 2020a; Wyser et al., 2020; Klein et al., 2022b; Cockx et al., 2023). If only SDCs are included as regressors in the GLM filter, the residual term ϵ in Equation (3) represents the SDC-cleaned data *Y*_*clean*_ (Klein et al., 2022b; Cockx et al., 2023). This can be calculated by rearranging the equation as follows:

$$\begin{array}{l} \begin{array}{c} \epsilon =Y-X_{SDC}\cdot \beta_{SDC}\\ =Y_{clean} \end{array} \end{array}$$

Here, *X*_*SDC*_ represents the design matrix containing only the SDCs, and β_*SDC*_ represents the estimated coefficients reflecting the influence of the SDCs on the uncorrected data *Y* (Klein et al., 2022b).

In contrast to the conventional application of the GLM, which uses SDCs (and/or other auxiliary signals) as (nuisance) regressors in addition to task-related regressors, this GLM filter approach is particularly suitable for real-time processing as it directly provides a corrected time series instead of statistical outcomes such as beta values (Klein et al., 2022b). Beyond SDCs, the inclusion of additional systemic physiological measures as nuisance regressors would be in line with the increasing recognition of their value in improving our understanding of brain-body interactions (Scholkmann et al., 2022). In this context, Scholkmann et al. (2022) emphasized that brain activity is strongly influenced by physiological changes and body interactions such as breathing and heart-brain connections, suggesting that it is too simplistic to view the brain in isolation. They proposed viewing the brain as integrated into the body and its environment and introduced a method called systemic physiology augmented fNIRS (SPA-fNIRS) to address this problem (Scholkmann et al., 2022). The goal of SPA-fNIRS is to combine fNIRS signals with systemic physiological signals that track cardio-respiratory and autonomic nervous system indicators such as arterial carbon dioxide levels, blood pressure, respiratory and heart rate, oxygen saturation, skin conductance and photoplethysmography. By integrating a variety of physiological data, SPA-fNIRS provides a nuanced analysis of the relationships between fNIRS signals and systemic physiology (Scholkmann et al., 2022). In this context, the previously mentioned tCCA approach (see Section 3.3) with SDCs alone or in combination with other auxiliary signals might be particularly suitable as an approach to generate optimal nuisance regressors (von Lühmann et al., 2020a; Scholkmann et al., 2022). This method takes into account the non-instantaneous and variable coupling between the fNIRS signals and auxiliary data (e.g., systemic physiology and SDCs) and has demonstrated its superior performance over the classic GLM-based approach using only SDCs (von Lühmann et al., 2020a) and therefore could be considered as a promising method for correction in real time. However, this approach has not yet been tested in a real-time scenario and it remains to be shown whether performance will improve in this case as well. SPA-fNIRS is also still in the development phase and faces challenges in implementation, particularly regarding the complexity and effort of adding multiple sensors, which may limit its applicability in certain experimental settings (Scholkmann et al., 2022). These limitations highlight the need for further development of both hardware and software to fully exploit the potential of SPA-fNIRS (Scholkmann et al., 2022), particularly for real-time applications.

Although the use of SDCs is considered the gold standard for extracerebral systemic artifact correction, not all researchers have access to SDCs, either due to limited availability or financial constraints. However, in such cases, alternative correction methods can be used instead (Pfeifer et al., 2018; Klein and Kranczioch, 2019; Santosa et al., 2020; Klein et al., 2022b). A simple alternative is to use a common average reference (CAR) filter (Bauernfeind et al., 2014; Klein et al., 2022b).

**Common Average Reference (CAR)**

Using CAR, a corrected version of each channel's signal *y*_*clean*_ is obtained by subtracting the average signal across all channels, or a predefined number, denoted as *c*, from its uncorrected version *y*:

$$\begin{array}{l} y_{clean}=y-\frac{1}{c}\cdot \overset{c}{\underset{i=1}{\sum }}y_{i} \end{array}$$

Instead of subtracting the averaged time course, an alternative approach could be to use the SSR method (as described in Equation 4) to regress the averaged signal across all or a predefined number of channels from each individual channel (Pfeifer et al., 2018). The CAR method offers simple and straightforward calculations and should therefore easily applicable in real-time preprocessing. However, it is important to note that the CAR method is susceptible to noise and may overcorrect the data (Bauernfeind et al., 2014; Klein et al., 2022b).

Another alternative method when SDCs are not available is to use a PCA (Abdalmalak et al., 2022) or a baseline PCA method (bPCA; Zhang et al., 2005; Santosa et al., 2020). PCA can be viewed as a spatial filter that reduces the dimensionality of a data set while maximizing the variance in the data (Jolliffe and Cadima, 2016; Fang and Boas, 2009). PCA converts the original set of variables into a new set of variables that represent linear combinations of the original variables, the PCs (Jolliffe and Cadima, 2016; Fang and Boas, 2009). The difference between PCA and bPCA lies in the data to which the algorithm is applied. In bPCA, the algorithm is applied to an individually collected baseline, while PCA is applied directly to the data to be corrected (Zhang et al., 2005; Santosa et al., 2020). The underlying assumption for this correction approach is that the systemic artifact accounts for a significant portion of the variance in the data and therefore should be captured by the initial set of PCs (Abdalmalak et al., 2022).

**Principal Component Analysis (PCA)**

For this approach, a singular value decomposition (SVD) is calculated based on either the spatial covariance matrix *C* of the data *Y* or the data itself (or the baseline in the case of bPCA):

$$\begin{array}{l} Y=U\Sigma V^{T} \end{array}$$

The SVD yields the matrix *V*, which represents the spatial information of the data, and the diagonal matrix Σ, which contains the singular values (i.e., a kind of “importance factors”) and the matrix *U* represents the temporal structure of the data (Zhang et al., 2005). The reconstruction of the cleaned signal *Y*_*clean*_ is then performed using the lower part of the spectrum of singular values (i.e., those corresponding to noise are removed):

$$\begin{array}{l} Y_{clean}=U_{k}\Sigma_{k}V_{k}^{T} \end{array}$$

where *k* represents the truncated versions of *U*, Σ, and *V*^*T*^, after removing the *k* largest singular values. Because the optimal value for *k* can vary between subjects, some researchers instead report the amount of variance to be retained (Cooper et al., 2012; Brigadoi et al., 2014).

However, since task-related brain activity should also contribute significantly to the overall variance, it is possible that the corresponding brain activity is present in the first *m* PCs. In contrast to traditional PCA, bPCA has the advantage of avoiding the risk of removing task-related information when the assumption that most of the variance is explained by artifacts is incorrect (Santosa et al., 2020). Overall, however, it should be noted that the assumption that the systemic artifacts explain the majority of the variance in the data is rather arbitrary and requires validation (Abdalmalak et al., 2022). Furthermore, the performance of (b)PCA relies on specifying the number of components or the desired explained variance in advance, without prior knowledge of the data. This aspect can be particularly challenging in real-time applications, as the optimal choice of *m* may vary depending on the subject or experimental conditions. Despite these limitations, (b)PCA remains a possible option for real-time fNIRS processing.

The global component removal (GCR) method proposed by Zhang et al. (2016) offers a similar correction procedure. In contrast to PCA, GCR also takes into account information about the (individual) channel locations (Zhang et al., 2016).

**Global Component Removal (GCR)**

The GCR method uses a matrix *D* to store the 3D channel positions. This matrix is then smoothed using a Gaussian kernel filter:

$$\begin{array}{l} G\left(D\right)=e^{\frac{-D^{2}}{2\sigma^{2}}} \end{array}$$

The width of the Gaussian kernel is defined by σ and has been found to be effective with σ = 46° (Zhang et al., 2017; Noah et al., 2021). After applying SVD to *Y*, the resulting vectors *v*_*i*_ in matrix *V* are then smoothed by convolving them with the Gaussian kernel *G*:

$$\begin{array}{l} v_{i}^{*}=v_{i}*G \end{array}$$

This operation results in the smoothed vectors $v_{i}^{*}$ in the matrix V^*^. Convolution with the Gaussian kernel *G*, removes localized neural patterns from the vectors and preserves the spatial information of the global component *Y*_*global*_ (Zhang et al., 2016). The *Y*_*global*_ can be computed by replacing *V* with V^*^ in the SVD formula:

$$\begin{array}{l} Y_{global}=U\Sigma V^{*} \end{array}$$

Finally, to obtain the cleaned data *Y*_*clean*_, the global component *Y*_*global*_ is subtracted from the original uncorrected data *Y*_*task*_ (Zhang et al., 2016):

$$\begin{array}{l} Y_{clean}=Y_{task}-Y_{global} \end{array}$$

The GCR method requires a relatively large kernel to achieve optimal performance. Therefore, to ensure the effectiveness of the GCR method, an optode coverage of at least 9 *cm*^2^ is recommended to avoid artificially negative brain activation (Zhang et al., 2016). Accordingly, the method should only be used when adequate head coverage can be ensured to minimize the risk of overcorrection and the resulting loss of brain activity information in the signal (Zhang et al., 2016; Klein et al., 2022b).

#### 3.5.3 Further considerations

**Figure 12:**
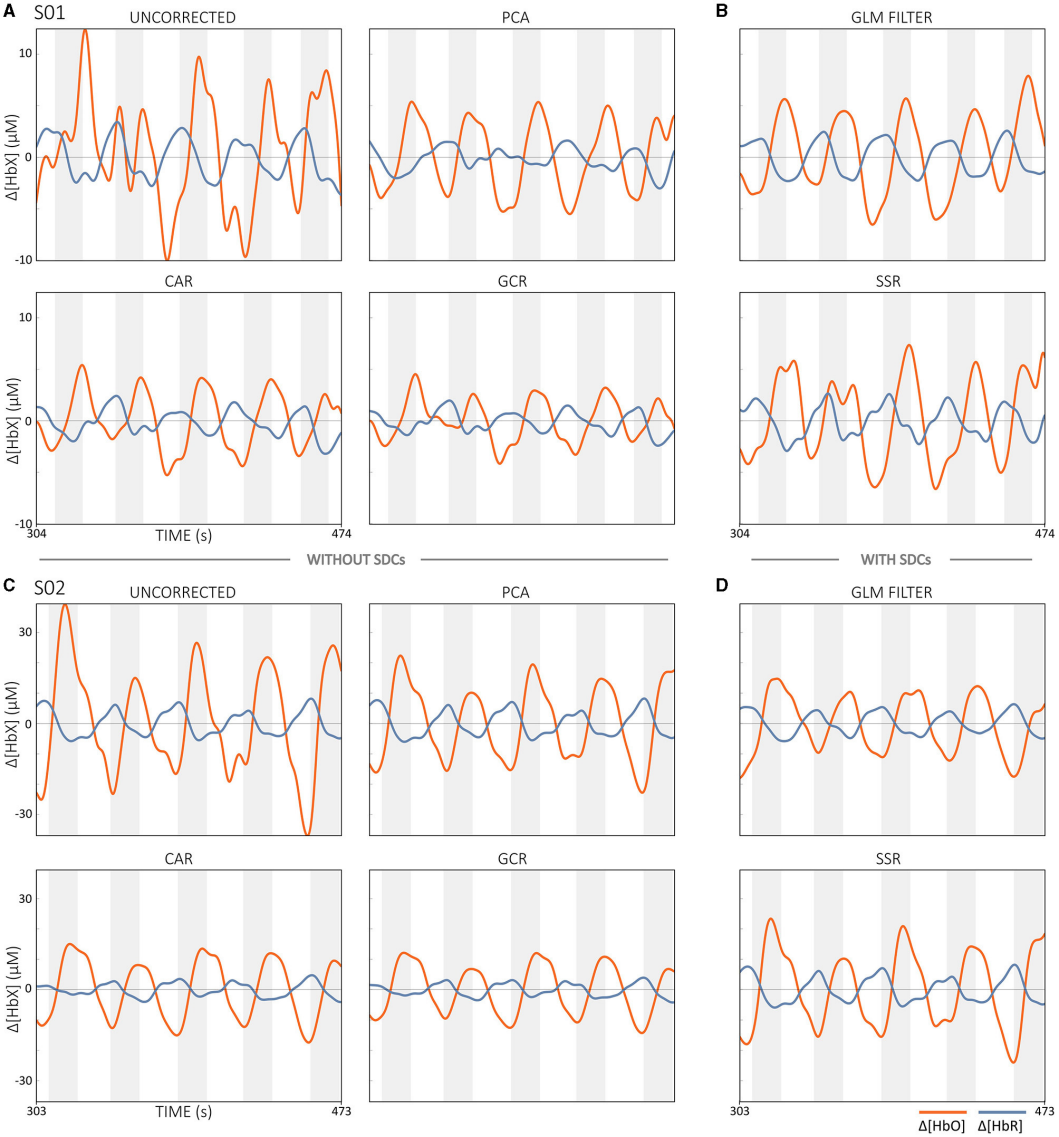
Illustration of the effects of extracerebral systemic artifact correction methods with and without SDCs on two subjects **(A, B)** s01, and **(C, D)** s02 that performed a motor execution task. The task-evoked time series are shown for preprocessed data without correction (UNCORRECTED). Correction methods without SDCs, including CAR, PCA, and GCR, as well as correction methods with SDCs, such as GLM-filter-based correction using eight SDCs (GLM FILTER) and SSR with the spatially closest SDC, are used to present the data corrected for extracerebral systemic artifacts. Gray areas indicate task periods.

According to Kohl et al. (2020), only a few NFB studies have attempted to correct for extracerebral systemic artifact contributions (e.g., using the CAR method; Hudak et al., 2017, 2018) and only one NFB study applied SDC-based correction in the real-time preprocessing pipeline (Fujimoto et al., 2017). Likewise, in BCI applications, only about 4% of studies have addressed this artifact (as of 2020; Von Lühmann et al., 2020b). As shown in Figure 12 and reported in previous studies, this type of artifact can significantly affect fNIRS signals, and also show different effects between subjects, tasks, brain regions and signal types, highlighting the importance of correction (Scholkmann et al., 2014; Tachtsidis and Scholkmann, 2016; Dravida et al., 2017; Santosa et al., 2020; Wyser et al., 2020; Klein et al., 2022b). The preferred choice for both offline and real-time corrections is probably to use SDCs. In particular, the GLM-based correction that includes all available SDCs for both the Δ[*HbO*] and Δ[*HbR*] data turned out to be the preferred choice, at least for the offline correction (Santosa et al., 2020; Wyser et al., 2020; Klein et al., 2022b). Additionally, it has been shown to be beneficial to include an additional regressor that represents the global component of this artifact, such as the first PC obtained from a PCA using all available SDCs (Wyser et al., 2020). If SDCs are not available, the CAR method or a regression-based approach using PCA or bPCA could be a potentially promising alternatives (Pfeifer et al., 2018; Santosa et al., 2020; Abdalmalak et al., 2022; Klein et al., 2022b).

Systematic validation of correction methods with and without SDCs, especially in real-time applications, is currently limited. Therefore, the question of which correction methods work best in real-time analysis cannot yet be answered. In any case, correction of extracerebral systemic artifacts is crucial for both offline and real-time fNIRS applications and should by no means be neglected as inadequate artifact correction can affect the interpretation of brain activity. In particular, it can either falsely mimic or mask the true underlying brain activity (Tachtsidis and Scholkmann, 2016), thereby compromising the accuracy and reliability of real-time systems. Accordingly, it is important to choose an appropriate correction approach to ensure the credibility and validity of the fNIRS data in all types of analyses.

## 4 Discussion

High spatial specificity and signal quality are essential for reliable and effective fNIRS-guided real-time applications such as NFB and BCIs. Spatial specificity can be crucial, particularly for repeated measurements, as it allows precise localization of relevant brain activity. In addition, signal quality could directly affect the reliability of the recorded neural signals, as only sufficient quality ensures robust and meaningful information extraction. These factors can play a crucial role in building user trust, thereby encouraging positive interactions with real-time technology. The aim of the present work was therefore to present and compare possible options that could contribute to improving spatial specificity and signal quality in the context of fNIRS-based real-time applications.

### 4.1 Improving spatial specificity

Despite the lower spatial resolution of fNIRS compared to fMRI, fNIRS offers the advantage of capturing spatially specific measurements of superficial cortical brain regions in more natural environments (Scarapicchia et al., 2017; Pinti et al., 2020; Klein et al., 2022a). However, challenges such as the typically limited number of available optodes and the lack of individual anatomical data can make precise targeting of specific ROIs difficult (Brigadoi et al., 2018; Zimeo Morais et al., 2018). Therefore, the development and use of standardized yet adaptable methods is particularly important.

**Figure 13:**
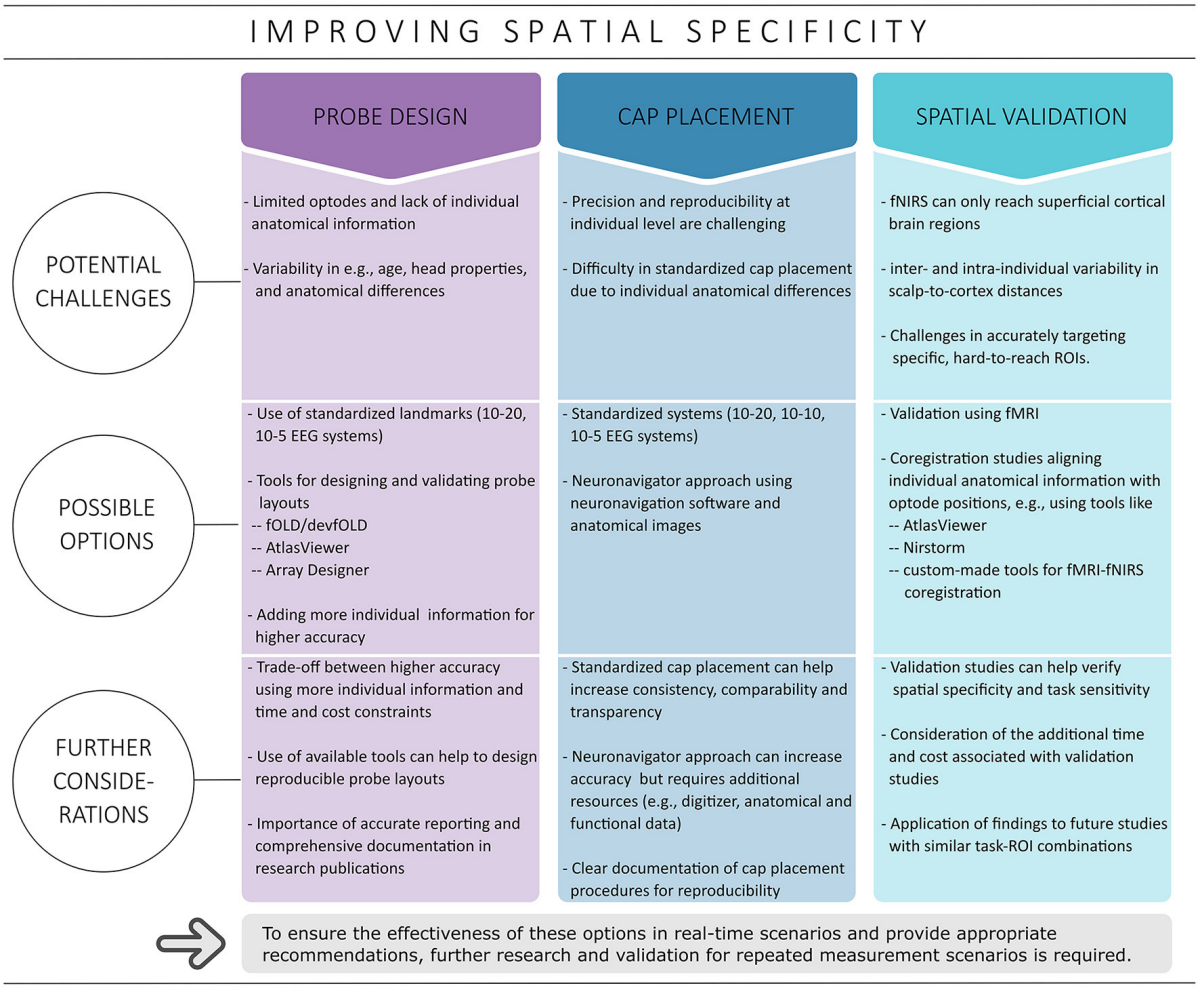
Tabular overview of all options discussed in this paper that could help improve spatial specificity, including potential challenges, possible options and further considerations. Please note that this overview does not claim to be complete and only represents suggestions.

The present work focused on three possible options to improve spatial specificity for (real-time) fNIRS experiments: probe design, precise cap placement, and systematic validation of fNIRS' ability to precisely target specific ROIs. With respect to optimizing probe design (e.g., Aasted et al., 2015; Brigadoi et al., 2018; Zimeo Morais et al., 2018; Fu and Richards, 2021) and in the development of methods for cap placement, remarkable progress has already been made (e.g., Oostenveld and Praamstra, 2001; Novi et al., 2020a; Wu et al., 2021). In addition, spatial validation techniques have been developed and applied in studies to assess the precision of fNIRS measurements in targeting specific brain regions and tasks (Toronov et al., 2001, 2003; Mehagnoul-Schipper et al., 2002; Strangman et al., 2002; Cui et al., 2011; Abdalmalak et al., 2017; Wagner et al., 2021; Klein et al., 2022a; Novi Junior et al., 2023; Pereira et al., 2023). However, despite these advances, a gap remains in the comprehensive documentation and validation of methods to improve spatial specificity in repeated real-time applications. To close this gap, the aim of this work was to provide an initial overview of possible options that have already been implemented in order to advance future standardization for real-time applications. A summary of all potential challenges, possible options, and discussed further considerations regarding their benefits for improving spatial specificity in fNIRS experiments is shown in Figure 13. Whether these methods actually improve the application of fNIRS in real-time scenarios remains an open question and requires future systematic validation.

### 4.2 Improving signal quality

The presence of various artifacts represents a significant challenge in fNIRS research in general (Pinti et al., 2020), and is particularly a problem in real-time applications, as uncorrected signals can cause the system to run on noise instead of real brain activity, reducing the reliability of these applications (Klein et al., 2022b). There have already been several efforts to improve offline (pre-)processing steps, including aspects such as channel quality assessment (Pollonini et al., 2014, 2016; Sappia et al., 2020), mBLL calculations (Scholkmann and Wolf, 2013; Scholkmann et al., 2014; Whiteman et al., 2017), temporal filtering (Pinti et al., 2019), MA correction (Scholkmann et al., 2010; Cooper et al., 2012; Brigadoi et al., 2014; Delgado Reyes et al., 2018; Jahani et al., 2018; Di Lorenzo et al., 2019; Fishburn et al., 2019; Von Lühmann et al., 2019; Novi et al., 2020b; Huang et al., 2022), and extracerebral systemic activity correction (Santosa et al., 2020; Von Lühmann et al., 2020b; Wyser et al., 2020; Noah et al., 2021; Abdalmalak et al., 2022; Klein et al., 2022b). Despite these advances, not all methods are suitable for real-time processing and real-time validation remains limited (Lotte et al., 2018; Klein et al., 2022b).

So how can we further address these challenges and leverage existing technologies and methodologies to advance the field of real-time fNIRS applications? For instance, Lotte et al. (2018) suggested that real-time rather than offline validations are essential for evaluating the performance of real-time methods. However, conducting multiple real-time tests for each algorithm and each individual presents logistical challenges due to time, cost, as well as individual and environmental variability. In this regard, simulated and semi-simulated data are invaluable for validating correction algorithms (von Lühmann et al., 2020a; Klein et al., 2022b) as they allow performance assessment against known ground truth data. Using semi-simulated data, a modeled HRF is combined with resting-state data, allowing recovery of the underlying HRF using selected correction methods while incorporating real physiological artifacts. Semi-simulated data could be used in combination with software tools like Turbo-Satori (Brain Innovations, Maastricht, The Netherlands; Lührs and Goebel, 2017), which allows for simulated real-time validation and integrates with MATLAB and Python, enabling even more advanced processing.

**Figure 14:**
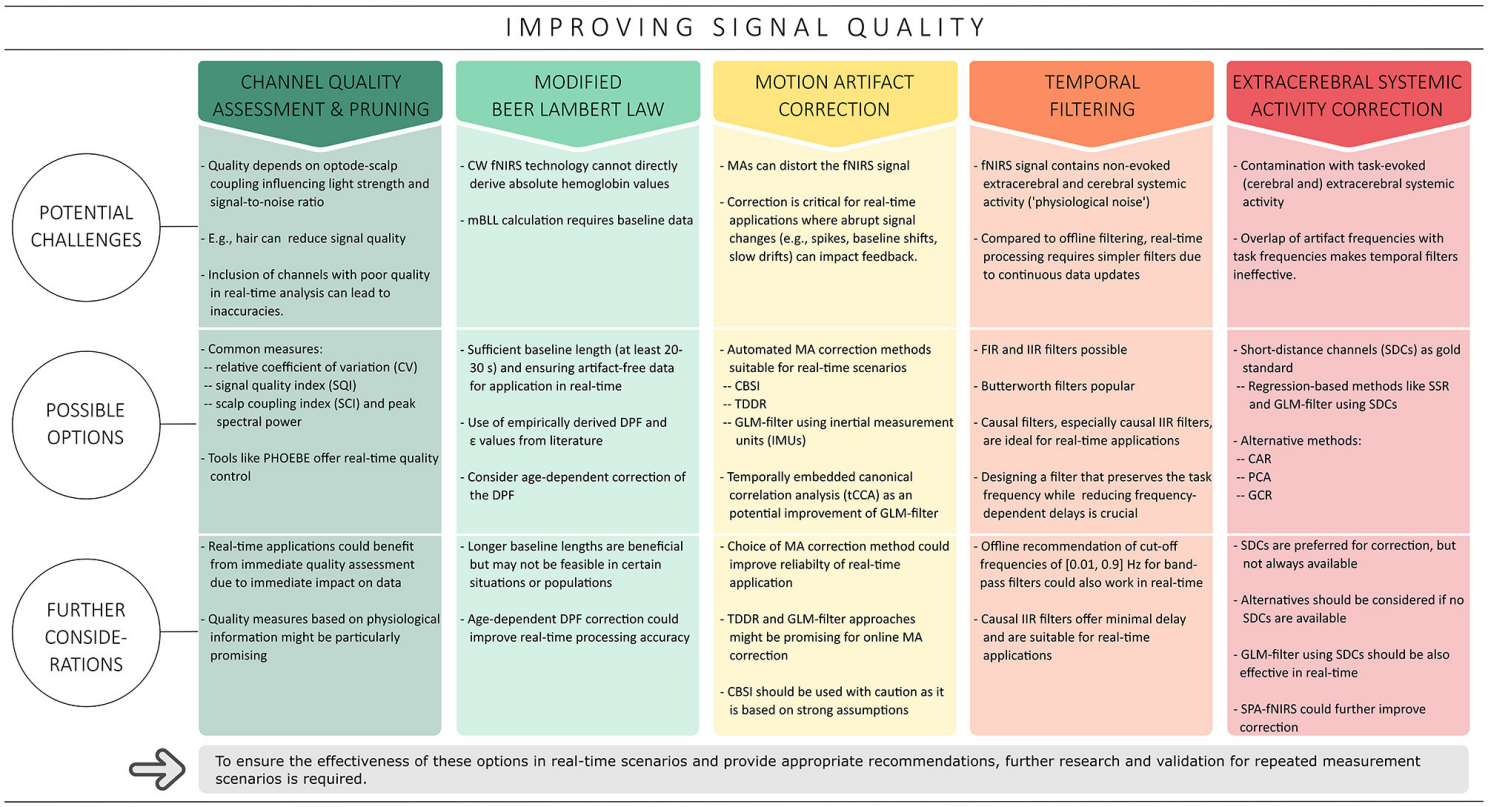
Tabular overview of all preprocessing steps discussed in this review that might help improve signal quality in real-time applications, including potential challenges, possible options and further considerations. Please note that this overview does not claim to be complete and only represents suggestions.

In summary, ensuring signal quality is critical in real-time applications such as BCI and NFB. Artifacts and noise can affect measurement reliability and could lead to possible incorrect interpretations and reduced user experience. Given the limitations of real-time data, which can not be post-corrected like offline data, immediate and accurate data processing is critical. Therefore, this review is intended to serve as a comprehensive resource for researchers working on fNIRS-based real-time applications by providing an overview of potential challenges, possible options, and further considerations on selected methods that could help improve signal quality. A summary of this can be found in Figure 14. As mentioned before in the spatial specificity section, this overview is mostly about documenting possible options and future work on systematic validation in real-time context is needed to make concrete recommendations. Please note that this review does not claim to be complete in terms of the selection of possible options, as it is based primarily on the author's subjective choice of methods and there may be additional options for improving signal quality for real-time applications.

### 4.3 Final remarks

While the replication crisis poses major challenges for all scientific disciplines, including fNIRS research, it also paves the way for (methodological) improvements (Poldrack et al., 2017; Paul et al., 2021; Yücel et al., 2021; Niso et al., 2022; Kelsey et al., 2023; Schroeder et al., 2023). However, rigorous study design, transparent reporting, and community collaboration are required to address this issue. Adopting best practices and open science principles, including study preregistration, creates a solid foundation for improving the reproducibility and credibility of (real-time) fNIRS studies (Yücel et al., 2021; Kelsey et al., 2023; Schroeder et al., 2023) and lays the foundation for progress in this direction. The purpose of this review was to contribute to these initiatives by providing a detailed overview of methods that could help to optimize real-time applications.

In addition to refining spatial specificity and signal quality, topics such as feature extraction and feedback algorithms should not be forgotten. Progress has already been made in these areas (e.g., Naseer and Hong, 2015; Paulmurugan et al., 2021). However, ever-evolving computing capabilities and advances in machine learning and artificial intelligence suggest that these assessments should be updated regularly, but this is beyond the scope of the present work.

The aim of the present review was to guide future advances in real-time applications by discussing potential challenges, exploring possible options, and providing further considerations that could help improve both spatial specificity and signal quality to advance research, strengthen existing applications and stimulate innovation in this rapidly evolving field.

## Author contributions

FK: Conceptualization, Investigation, Methodology, Visualization, Writing – original draft, Writing – review & editing, Project administration.

## Footnotes

^1^see e.g., Discover Article, 2019 (accessed 9 April 2024); University of Tübingen press release (in German), 2019 (accessed 9 April 2024).
